# Phylogenetic prioritization of HIV-1 transmission clusters with viral lineage-level diversification rates

**DOI:** 10.1093/emph/eoac026

**Published:** 2022-07-22

**Authors:** Rachel L Miller, Angela McLaughlin, Richard H Liang, John Harding, Jason Wong, Anh Q Le, Chanson J Brumme, Julio S G Montaner, Jeffrey B Joy

**Affiliations:** Molecular Epidemiology and Evolutionary Genetics, British Columbia Centre for Excellence in HIV/AIDS, Vancouver, Canada; Bioinformatics Program, University of British Columbia, Vancouver, Canada; Molecular Epidemiology and Evolutionary Genetics, British Columbia Centre for Excellence in HIV/AIDS, Vancouver, Canada; Bioinformatics Program, University of British Columbia, Vancouver, Canada; Laboratory Program, British Columbia Centre for Excellence in HIV/AIDS, Vancouver, Canada; Vancouver Coastal Health, Vancouver, Canada; Clinical Prevention Services, British Columbia Centre for Disease Control, Vancouver, Canada; Laboratory Program, British Columbia Centre for Excellence in HIV/AIDS, Vancouver, Canada; Laboratory Program, British Columbia Centre for Excellence in HIV/AIDS, Vancouver, Canada; Department of Medicine, University of British Columbia, Vancouver, Canada; Department of Medicine, University of British Columbia, Vancouver, Canada; British Columbia Centre for Excellence in HIV/AIDS, Vancouver, Canada; Molecular Epidemiology and Evolutionary Genetics, British Columbia Centre for Excellence in HIV/AIDS, Vancouver, Canada; Bioinformatics Program, University of British Columbia, Vancouver, Canada; Department of Medicine, University of British Columbia, Vancouver, Canada

**Keywords:** molecular epidemiology, HIV, transmission clusters, phylogenetics

## Abstract

**Background and objectives:**

Public health officials faced with a large number of transmission clusters require a rapid, scalable and unbiased way to prioritize distribution of limited resources to maximize benefits. We hypothesize that transmission cluster prioritization based on phylogenetically derived lineage-level diversification rates will perform as well as or better than commonly used growth-based prioritization measures, without need for historical data or subjective interpretation.

**Methodology:**

9822 HIV pol sequences collected during routine drug resistance genotyping were used alongside simulated sequence data to infer sets of phylogenetic transmission clusters via patristic distance threshold. Prioritized clusters inferred from empirical data were compared to those prioritized by the current public health protocols. Prioritization of simulated clusters was evaluated based on correlation of a given prioritization measure with future cluster growth, as well as the number of direct downstream transmissions from cluster members.

**Results:**

Empirical data suggest diversification rate-based measures perform comparably to growth-based measures in recreating public heath prioritization choices. However, unbiased simulated data reveals phylogenetic diversification rate-based measures perform better in predicting future cluster growth relative to growth-based measures, particularly long-term growth. Diversification rate-based measures also display advantages over growth-based measures in highlighting groups with greater future transmission events compared to random groups of the same size. Furthermore, diversification rate measures were notably more robust to effects of decreased sampling proportion.

**Conclusions and implications:**

Our findings indicate diversification rate-based measures frequently outperform growth-based measures in predicting future cluster growth and offer several additional advantages beneficial to optimizing the public health prioritization process.

## BACKGROUND AND OBJECTIVES

**Figure 1.**
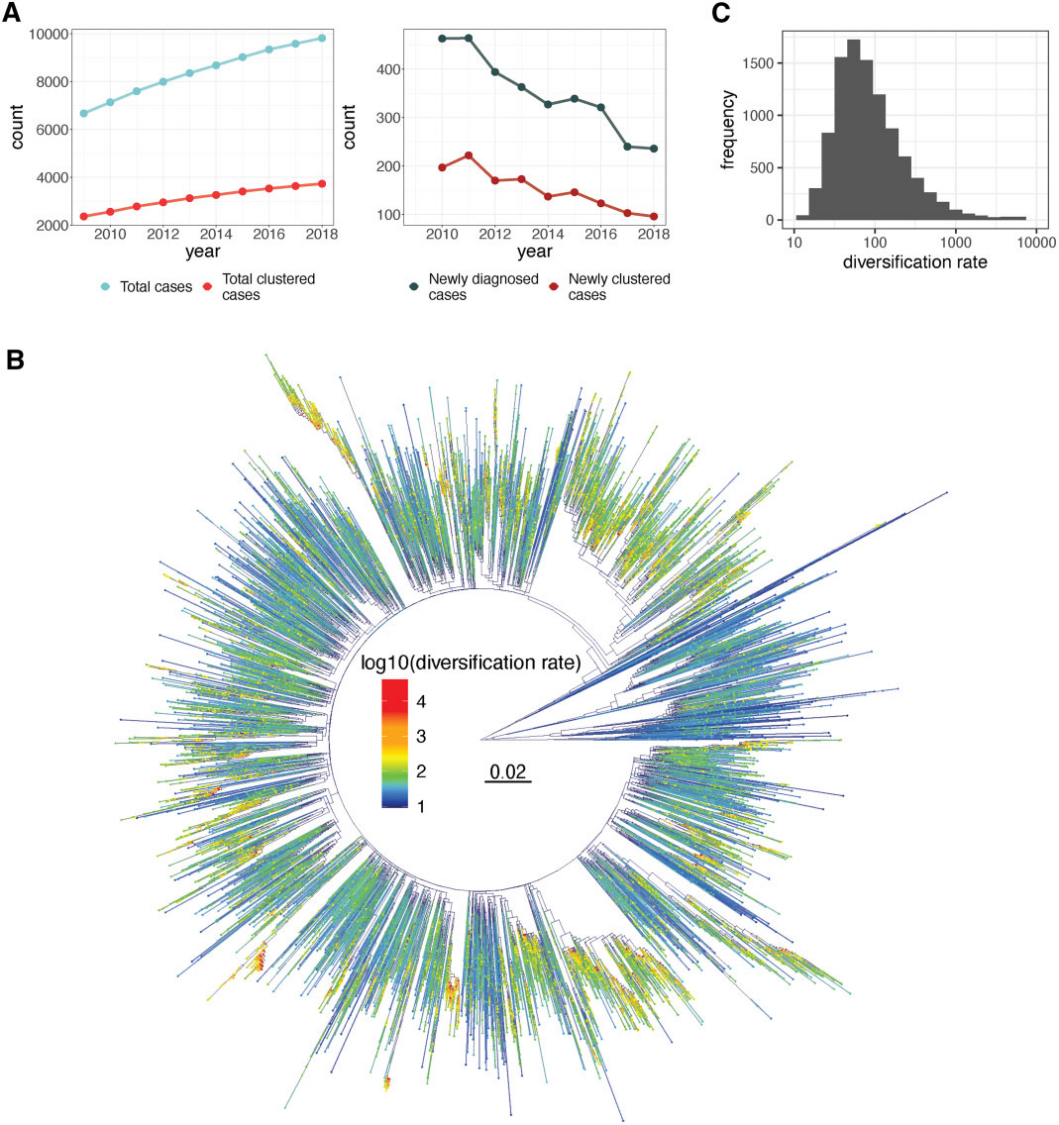
Diversification rate distribution, phylogeny and clustering over time. (**A**) Change in total cases, total clustered cases, newly diagnosed cases and newly clustered cases over time. Although total cases and total clustered cases are increasing over time, the yearly numbers of newly diagnosed cases and cases joining clusters have been consistently declining since 2011. (**B**) 95% majority-rule consensus tree for 2018, with tips and pendant edges coloured by lineage-level diversification rate. Warmer colours and thicker edges highlight high diversification rates, suggesting rapid transmission. (**C**) The distribution of median lineage-level diversification rates across the 100-replicate tree distribution for all individuals. The right-tailed distribution indicates that stratifying by diversification rate is likely to be effective in distinguishing a small top-priority population of individuals

Current methods of HIV-1 outbreak management in countries with sufficient resources and government support to sustain continuous monitoring are highly effective [1–4]. In the province of British Columbia (BC), Canada, near real-time phylogenetic monitoring of the local HIV-1 epidemic has been employed since 2013 to detect and prioritize groups with the highest risk of transmission for focused public health intervention [5]. These focused interventions have allowed limited public health resources to be allocated with greater efficiency and 8 years of using this method to track and respond to the epidemic have contributed to substantial decreases in new HIV diagnoses [6, 7]. Decreasing diagnoses are likely also driven in part by improved access to antiretroviral therapy (ART), the expansion of harm reduction strategies [8], and increased uptake of pre-exposure prophylaxis (PrEP) following the 2018 decision to provide PrEP to eligible individuals at no cost [9]. Additionally, phylogenetic monitoring has allowed fine-grained inferences about trends in the BC epidemic, such as the number of new cases joining clusters over time, a metric quantifying local transmission shown by our dataset to have consistently declined over the past 8 years (Fig. 1A). The strategies employed in BC have not only resulted in a more rapid decline in cases relative to other Canadian provinces [10], but have also helped BC make exceptional progress towards the UNAIDS 95-95-95 targets [6, 11]. Although the methods used in BC are highly effective, networks of transmission still exist and more comprehensive characterization and prioritization of transmission groups could improve epidemic control through more focused action.

Phylogenetic monitoring of HIV-1 transmission is highly amenable because viral populations are known to diverge following transmission due to the rapid rate of evolution of HIV and host-specific selection [12, 13]. Viral genetic divergence can be quantified using patristic distance, the sum of the branch lengths linking two nodes in a phylogenetic tree. A pairwise patristic distance cutoff can delineate individuals into groups based on the genetic similarity of the viruses they harbour, set such that these ‘transmission clusters’ represent groups of infections likely to be related by recent transmission events [5]. Furthermore, the sequence data required for these analyses are often readily available as a byproduct of routine drug resistance genotyping. Importantly, due to the uncertainty introduced by incomplete sampling of the infected population, phylogenetic methods can never be used to definitively infer transmission directionality between two individuals or cluster members. Once a set of transmission clusters has been identified, they can be prioritized such that limited public health resources can be allocated most rapidly to clusters with the greatest potential for future growth, in order to quickly quell the greatest number of transmissions before shifting focus to less urgent clusters.

Prioritized clusters may receive different types of public health attention depending on their characteristics. Linkage or re-linkage to care can help begin or improve adherence to ART, optimize ART regimen choices, and encourage consistent viral load suppression. Contacts of cluster members at risk for transmission can also be offered targeted PrEP and urged to seek diagnostic testing. Both cluster members and their contacts can be encouraged to maintain safe practices regarding their risk behaviours, such as use of condoms or safe injection materials. By employing a combined approach tailored to cluster characteristics, public health teams in BC have previously been able to notably reduce transmission associated with prioritized clusters [14].

Recently, the use of non-phylogenetic measures, either in combination with phylogenetic clustering [5, 15], non-phylogenetic clustering [1, 2, 16] or without directly considering clustering [17] have been proposed to enhance prioritization processes. The addition of quantitative measures such as cluster size, previous cluster growth or mean cluster viral load can make the prioritization procedure less subjective. However, there are no established cutoffs defining when such measures reach a level warranting rapid intervention, nor are there established methods for estimating the combined effects of multiple measures. Consequently, cluster prioritization processes remain heavily reliant upon local expertise and subjective interpretation and are thus limited in their ability to be consistent and widely applicable. Furthermore, use of measures such as previous cluster growth or average cluster viral load rely on data collected in previous years or additional linked data, both of which may not always be available.

In this study, we employ both empirical and simulated data to evaluate the hypothesis that cluster prioritization measures based on phylogenetically derived lineage-level diversification rates will perform at least equally as well as commonly used non-phylogenetic growth-based prioritization measures in stratifying clusters based on transmission activity, while operating independently from supporting meta data and remaining free of need for subjective or historical interpretation. We use BC empirical data to compare phylogenetic diversification rate-based measures to growth-based measures in their ability to separate clusters labelled by BC public health protocol as ‘priority’ from the remaining clusters. We further compare prioritization measure performance through a simulation study to reduce the effects of biases that hinder empirical data, such as incomplete sampling and differential cluster intervention. Thus, simulation facilitates direct comparison of the relationship between each prioritization measure and future cluster growth, as well as the direct number of transmissions resulting from prioritized clusters.

## METHODOLOGY

### Dataset

In BC, routine clinical care following every new HIV diagnosis includes drug resistance genotype testing performed by the BC Centre for Excellence in HIV/AIDS (BC-CfE). Viral RNA extracted from patient blood samples is used to determine which antiretroviral drugs will be most effective for each individual based on observed viral surveillance drug resistance mutations (SDRMs) [18]. After this information is reported back to the primary care clinicians, the BC-CfE retains both the HIV sequence data and associated metadata. As this process has been part of routine clinical care since 1996, it has led to the creation of a comprehensive dataset representing > 75% of the prevalence of HIV in BC [11]. The study dataset was restricted to data collected by the BC-CfE between May 1996 and December 2018 inclusive, totalling 35 752 partial pol HIV sequences derived from viruses sampled from 9822 patients. All data were doubly de-identified prior to analysis. The University of British Columbia—Providence Health Care Research Ethics Board granted ethical approval for this study (H07-02559).

### Phylogenetic analyses

All 35 752 anonymized sequences were aligned with MAFFT v7.310 [19]. Known SDRM codons were masked before inference of a distribution of 100 phylogenetic tree replicates using FastTree v2.1.10 [20]. In order to create trees reflective of the data available in previous years, the full trees were pruned using the ape R package [21] to create 10 subtrees containing the sequences sampled by the end of each year in the 2009–2018 period. Each subtree was rooted using the residual-mean-squared function in TempEst v1.5.3 [22] and pruned to contain only the earliest sequence available for each individual, leaving 9822 sequences in the largest trees. Transmission clusters were inferred from the full 2018 trees using a patristic distance threshold of 0.02 nucleotide substitutions/site, as described by Poon *et al*. [5]. Two individuals were considered linked if the phylogenetic patristic distance between their sequences was below the 0.02 substitution/site threshold in at least 50% of the 100-replicate tree distribution. Cluster membership was determined by considering these thresholds across all individuals, with the caveat that a cluster must contain at least five individuals before being defined as such in order to maintain confidentiality beyond double anonymization of the data. Lineage-level diversification rates were calculated for all tips in all trees and the median value associated with each tip in each year was kept for use in downstream analyses. In order to quantify the distribution of diversification rates for tips across the 100 trees, the median difference in diversification rate between a tip in a given tree and the same tip in all other trees was calculated. In general, the median difference in diversification rate across trees was very small, although some outlier tips did show greater variation (Supplementary Fig. S1). A 95% majority-rule consensus tree generated from the 2018 trees was generated in DendroPy v4.5.2 [23] and used to visualize median diversification rates across the 100-replicate tree distribution for each tip (Fig. 1B).

The diversification rate-based measures assessed include mean (MeanDR), median (MedianDR), maximum (MaxDR) and most recent diversification rate (MostRecentDR), as well as the mean of the top three (Top3MeanDR) and top five diversification rates (Top5MeanDR) in a cluster. Growth-based measures assessed include previous year (PrevYrGrowth), 3-year (Prev3YrGrowth) and 5-year growth (PrevYrGrowth), as well as cluster size (ClustSize), previous year cluster growth squared (ClustGrowthSq) and whether or not more than five cases have joined a cluster in the previous year (PrevYr5CasePlus). ClustGrowthSq and PrevYr5CasePlus were included for the purpose of comparison to previous studies [16, 24]. Cluster growth was calculated by subtracting the number of sampled cluster members at the beginning of a given growth period from the number of cluster members sampled at the end of that growth period.

### Lineage-level diversification rate

Lineage-level diversification rate is a phylogenetically derived measure first described by Jetz *et al*. [25] that tracks the historical branching rate of tips in the phylogeny. In an epidemiological context, because a new lineage is formed following transmission to a new host, viral lineage-level diversification rate thus serves as a proxy for transmission rate. Lineage-level diversification rate accounts for both the number of branching events and the branch lengths in a phylogenetic tree, and as a tip-weighted measure, it emphasizes recent events. Tips sharing very recent common ancestry with many other lineages will have higher diversification rates, providing evidence for rapid transmission. The opposite is true for tips existing in areas of the tree where fewer lineages stem from fewer recent branching events—these tips will have lower diversification rates, suggesting slower transmission rates, or possibly poorer sampling. All mentions of ‘diversification rate’ used in this work specifically refer to lineage-level diversification rate.

### Evaluation of growth-based versus diversification rate-based measures: BC data

The ability of growth-based measures and diversification rate-based measures to stratify transmission clusters based on transmission activity was evaluated based on the ability of each measure to separate clusters marked as ‘priority’ by the BC public health protocol in a given year from the remainder. Prioritization decisions made by the BC protocol can be a result of rapid cluster growth, rapid symptom onset seen in one or more cluster members, or combinations of other factors recognized by the public health team as concerning. Successful distinction between these two groups of transmission clusters was assessed using Mann–Whitney *U* tests.

### Epidemic simulation

The need for simulation of the BC epidemic is motivated by two factors. First, intervention decreasing transmission would be expected to reduce lineage-level diversification rate for a transmission cluster, meaning that clusters known to be consistently labelled as priority may display decelerating diversification rates despite having high potential for transmission. Second, evaluating novel methods using the set of labels defined by the BC public health protocol as a benchmark assumes that this method accurately captures the true set of clusters with the greatest potential for transmission, which may not necessarily be the case. Simulated epidemics allow evaluation under circumstances where intervention is not applied differentially between clusters and the true clusters with the greatest potential for propagating future transmissions are known.

Simulation was performed using the FAVITES framework [26] to create a contact network, seed infected individuals, generate transmission events and the viral evolution between them, and subsequently sample viral sequences. Simulations were set to run over a 10-year period beginning in 2009, with a base set of parameters adapted from Moshiri *et al*. [26] and six variations of this set designed to encapsulate a range of reasonable matches to the characteristics of the BC epidemic while still containing enough variation in epidemic parameters to retain generalizability to epidemics in other locations. Full description of parameter selection can be found in the Supplementary Materials.

### Evaluation of growth-based versus diversification rate-based measures: simulated data

For each replicate of each of the seven parameter sets, simulated sequences spanning the full 10-year simulation period were aligned in MAFFT and phylogenies were inferred with FastTree. After rooting on all branches via LSD2 [27], each phylogeny was pruned to 10 subtrees, each representing the data collected by the end of a given simulation year. Transmission clusters were inferred for each subtree and lineage-level diversification rates were calculated for all tips. Cluster-level prioritization measures were calculated for each subtree such that the Spearman correlation between a prioritization measure in a given year and the amount of cluster growth in a given future year or period of years could be determined and summarized across replicates. For each year of prioritization measure calculation, cluster growth was measured across two different growth periods: next-year growth and total future growth. Next-year growth was defined as the number of individuals who joined a cluster in the year following prioritization measure calculation. Total future cluster growth describes the number of individuals who joined a cluster across the remaining years in the period of study after the year of prioritization measure calculation. For example, for prioritization measures determined in 2009, total future growth describes how many individuals joined the cluster between 2010 and 2018. Additionally, clusters were ranked by each prioritization measure and individuals from the top clusters (up to inclusion of 100 individuals, or the size of the top cluster, if its size exceeded 100) were used to determine the number of direct resulting transmissions, for comparison to the number of transmissions produced by a random sample of lower-ranked clusters containing the same number of individuals.

## RESULTS

### BC HIV transmission clusters

The numbers of both clustered and non-clustered cases decreased over time, as did the number of new cases (Fig. 1A). In more recent years, a larger proportion of the new cases being diagnosed were non-clustered, further suggesting that more sensitive methods will be required to appropriately address all cases going forward. However, clusters capture pockets of closely related transmissions, and as comparison of diversification rates from un-clustered versus clustered tips suggests (Supplementary Fig. S2), the potential for rapid transmission is significantly greater within clusters (Mann–Whitney *P* < 0.001), thus rationalizing the focus on these groups as primary public health targets. Even within clusters, hundreds of cases occur each year that could be prevented if the accuracy and resolution of prioritization was increased. The right-tailed log distribution of diversification rates highlights the ability of this measure to allow more focused prioritization, as there are very few diversification rates that are very high relative to the majority of the measurements (Fig. 1C).

### Growth-based versus diversification rate-based measures: BC data

**Figure 2.**
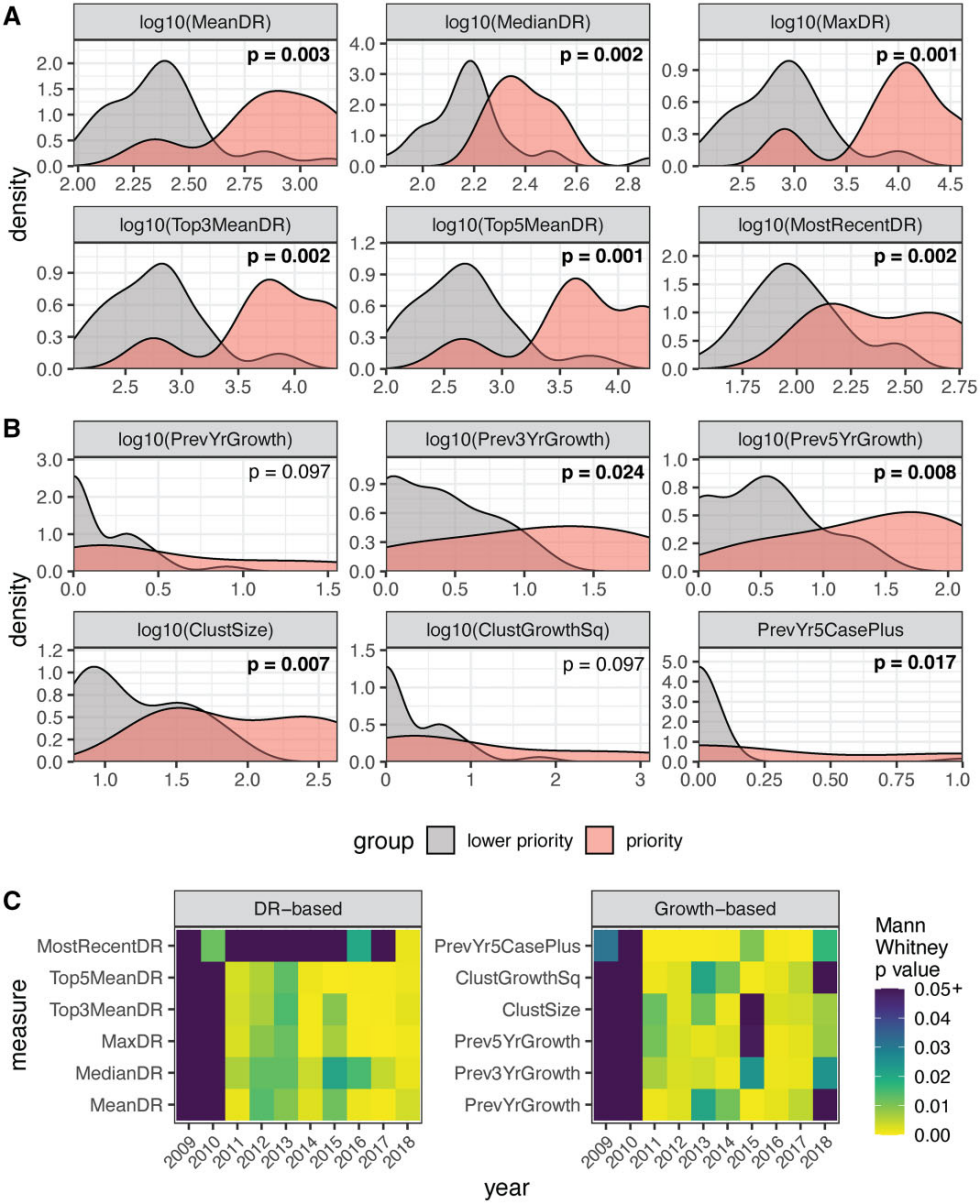
Comparison of prioritization measures between priority and non-priority groups, using empirical data. Density plots showing the difference in (**A**) diversification rate-based measures (DR-based) in 2018 or (**B**) growth-based measures in 2018 between clusters defined as ‘priority’ by the current public health protocol for immediate intervention and the remainder of the clusters to next be addressed, marked here for the purpose of comparison as ‘lower priority’. Only clusters that had newly diagnoses cases added in the previous year are shown. Infinite values created by the log10 transformation were forced to 0 for visualization purposes. DR-based measures frequently create clearer visual distinction between priority and lower priority populations. (**C**) Heatmaps of significance (as per Mann–Whitney *U* tests) of the separation between priority and lower priority groups, as produced by the measures we examined. *P*-values of 0.05 or higher are shown in purple. Rows represent different measures, and columns represent years. Diversification rate-based measures show similar consistency to growth-based measures in their ability to reveal a statistically significant difference between top priority and lower priority clusters across the 10-year period investigated

Some growth-based measures and some diversification rate-based measures were effective in distinguishing priority from non-priority clusters, as labelled by the current BC public health protocol. Using the year 2018 as a representative example, all six of the diversification rate-based measures tested showed a significant difference between priority and non-priority clusters (Mann–Whitney *P* = 0.0033, 0.0016, 0.00086, 0.0019, 0.0011, 0.0016). All tested measures except for the MostRecentDR created two clearly distinct peaks between the two groups of clusters, with the most obvious visual distinction between populations occurring for MeanDR, MaxDR, Top3MeanDR and Top5MeanDR (Fig. 2A).

In 2018, four of the six growth-based measures tested (Prev3YrGrowth, Prev5YrGrowth, ClustSize and PrevYr5CasePlus) showed a significant difference between priority and non-priority clusters (Mann–Whitney *P* = 0.024, 0.076, 0.0073, 0.017), although none of these four demonstrated clearly defined separation between the two populations of clusters (Fig. 2B).

Using these four measures consistently generates low values for low priority clusters, but values for the high priority clusters frequently form a wide distribution, making the two populations of clusters difficult to distinguish (Fig. 2B).

Assessing the tested measures over a 10-year period illuminated differences in consistency between measures and highlighted need for longitudinal testing of novel prioritization measures. Although many of the measures were relatively consistent over time, none achieved statistical separation in all 10 years (Fig. 2C) and visual separation between the populations varied (not shown). The most consistently effective growth-based measure, PrevYr5CasePlus, creates statistical separation in 9 years, but never creates two clearly visually distinct population peaks, possibly due to its binary nature (Supplementary Fig. S3). The next most consistent growth-based measure, Prev3YrGrowth, creates statistical separation in 8 years and provided somewhat clearer separation between the populations, particularly in 2016 (Supplementary Fig. S3H), suggesting that this may be the best growth-based candidate. However, some diversification rate measures (MeanDR, MaxDR, Top3MeanDR, Top5MeanDR) offer clearer visual separation between the populations in 2011–17, while still achieving statistical separation in 8 years. The separation between the two populations created by Top3MeanDR and Top5MeanDR weakens slightly in 2012–14 (Supplementary Fig. S3D–F), but these measures still create more obvious distinction than the growth-based measures during this period. Overall, we find diversification rate-based measures to be at least similarly effective to growth-based measures in differentiating the two populations, while potentially offering clearer boundaries.

### Growth-based versus diversification rate-based measures using simulated data

Phylogenies inferred from simulated data further demonstrated the strength of the relationship between phylogenetic diversification rate-based prioritization measures and future cluster growth, while also revealing differences in this relationship between the two groups of prioritization measures as the cluster growth period is extended.

**Figure 3.**
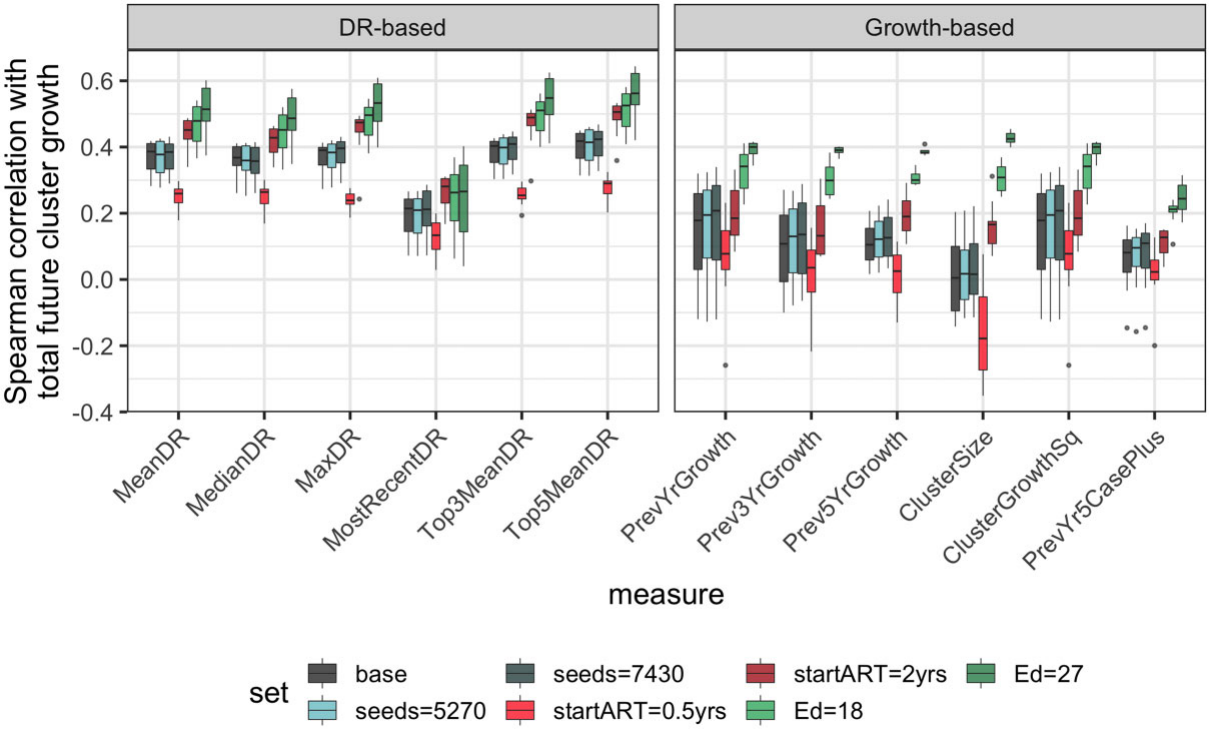
Comparison of correlation between prioritization measures and total future growth, using simulated data. Each box represents the median and IQR of the Spearman correlation between a given prioritization measure and parameter set combination and total future cluster growth from each simulation year to the end of the simulation period, across all growth periods from 2009 to 2018. The tested parameter sets include variations from the base set that differ in number of seed individuals, time between infection and the initiation of ART (startART) and the expected degree (Ed) of connectedness within the contact network. All other abbreviations are as defined in Fig. 2. In general, diversification rate-based measures showed stronger correlations with total future cluster growth than growth-based measures

In general, across parameter sets, the correlation between a prioritization measure and cluster growth in the next year was slightly stronger for diversification rate-based measures than for growth-based measures (median Spearman *r* = 0.34 vs 0.29; Supplementary Fig. S4). The difference between the two groups of prioritization measures becomes more striking when considering total future growth of a cluster across the remainder of the simulation period, with the diversification rate-based measures clearly showing stronger correlations across all parameter sets (median Spearman *r* = 0.39 vs 0.17; Fig. 3). Downsampled datasets revealed that as sampling proportion decreases to 75%, 50% and 25%, the positive relationship between diversification rate-based measures and growth in the next year weakens and, in some cases, becomes almost non-existent (median Spearman *r* = 0.15, 0.08, 0.04), suggesting that these measures are limited in the benefit they can confer in heavily under-sampled epidemics (Supplementary Fig. S5). However, the correlation between growth-based measures and next year growth not only experiences weakening in response to downsampling, but also reversal (median Spearman *r* = –0.15, –0.21, –0.35; Supplementary Fig. S5), meaning that without careful interpretation, growth-based measures have the potential to become actively misleading in a poorly sampled epidemic.

The relationships with total future growth in the remainder of the simulation period as sampling proportion decreases are more robust for both groups of measures (Supplementary Fig. S6). Diversification rate-based measures demonstrate very little change in correlation with future growth as sampling proportion is decreased (median Spearman *r* = 0.41, 0.40, 0.33), and although some growth-based measures such as PrevYrGrowth again show inverted correlations, other measures such as ClustSize and ClustGrowthSq retain a relatively similar effect size, particularly when the time to start ART or the number of contacts is increased from the base parameter set (Supplementary Fig. S6).

Considering a longer time period of cluster growth also reveals opposing temporal trends in the two groups of measures. Comparing the correlations between prioritization measures and next year growth versus total future growth revealed that effect sizes for diversification rate-based measures strengthened as the length of the cluster growth period was extended, but the opposite trend was seen for growth-based measures (Supplementary Fig. S7), suggesting that diversification rate-based measures may have a stronger relationship with long-term cluster growth. Furthermore, as the starting year for the cluster growth period becomes closer to the present, the strength of correlation for diversification rate measures remains relatively stable, but the strength of correlation for growth-based measures weakens slightly (Supplementary Fig. S7). Downsampled analyses showed that as sampling proportion decreases, the overall relationship between future growth and diversification rate-based measures weakens, but the increasing trend in effect size as the cluster growth period is extended is maintained (Supplementary Fig. S8). The weakening of this relationship is notably smaller for the total cluster growth periods than for those including only the next year after prioritization. The relationship between growth-based measures and future growth also weakens as sampling proportion decreases, but with the greatest decrease occurring for the next-year periods, such that the previous trend of weakening correlations as the growth period extends is reversed. As observed with diversification rate measures, the relationship between future growth and growth-based measures was also most robust to decreasing sampling proportion when considering total future growth (Supplementary Fig. S8).

Differences in the relationship between prioritization measures and future growth also exist across parameter sets. Regardless of growth period, both diversification rate and growth-based measures show much weaker correlations with cluster growth when the time to start ART is reduced to 0.5 years (Fig. 3; Supplementary Fig. S9). Conversely, as the time to start ART is increased to 2 years, correlations strengthen, even relative to the base parameter set. This trend continues even further when the average level of connectedness of individuals within the contact network increases. Additionally, when the time to start ART is increased or the expected degree of connectivity is increased, correlations with total future growth are more robust to downsampling. Together, these results suggest that prioritization measures as a whole may be at their most useful in epidemics with longer delays in connection to care, more connected at-risk populations and more barriers to treatment and prevention.

**Figure 4.**
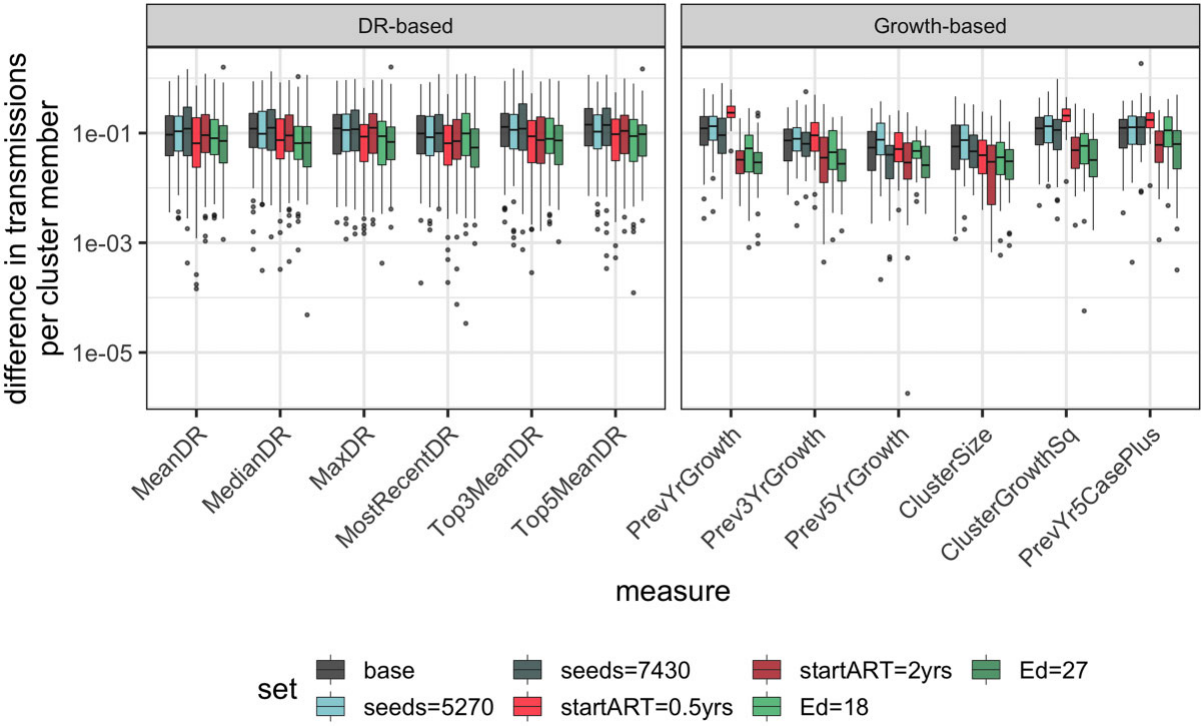
Difference in downstream transmissions across all prioritization measure and parameter set combinations. Each box represents the median and IQR of the difference in mean direct transmissions per cluster member between the top prioritized clusters, up to inclusion of 100 individuals (or the size of the top cluster, if its size exceeds 100), according to a given prioritization measure and a random sample of lower-ranking clusters containing the same number of individuals, across all clusters. The difference in mean direct transmissions was, in general, slightly higher for diversification rate-based measures than for growth-based measures, with the exception of previous year cluster growth in some parameter sets. Abbreviations are as defined in Figs 2 and 3

Analysis of the difference between the number of transmissions resulting from top-ranking prioritized clusters and a random sample of lower-ranking clusters containing the same number of individuals demonstrated additional benefits of using diversification rate-based measures. Without adjusting for cluster size, the difference in direct transmissions stemming from prioritized versus not prioritized clusters was higher for all growth-based measures (except PrevYrCasePlus), unless time to start ART was 0.5 years (median 4.1 vs 7.1, Mann–Whitney *P* < 0.001). However, when the number of direct transmissions was adjusted for current cluster size, the difference in direct transmissions prioritized by diversification rate measures was significantly larger (median 0.032 vs –0.023, Mann–Whitney *P* < 0.001) in the majority of parameter sets, with a notably larger disparity for parameter sets with increased time to start ART or contact network connections (Fig. 4). The shift in outcome after adjusting for cluster size perhaps suggests that diversification rate-based measures are more likely to prioritize smaller transmission clusters, capturing individuals with potential for rapid transmission that may otherwise go undetected until the cluster undergoes an amount of growth large enough to result in growth-based prioritization. Indeed, median cluster sizes were significantly smaller (Mann–Whitney *P* < 0.001) for clusters prioritized by diversification rate measures (median size = 41) than for clusters prioritized by growth-based measures (median size = 99; Supplementary Fig. S9), with the exception of PrevYr5CasePlus, which is biased by the fact that cluster initiation begins with a jump in size from zero to five. Diversification rate measures also showed greater consistency across parameter sets, indicating their ability to retain relevance in a wider range of scenarios. Downsampling demonstrated a similar trend to that of previous analyses, showing that decreases in sampling proportion generally result in maintenance of difference in transmissions for diversification rate measures (with the exception of one parameter set at 25% sampling) while the growth-based measures undergo a decline (median difference in transmissions = 0.16, 0.16, 0.13 vs 0.13, 0.12, 0.08; Supplementary Fig. S10).

## CONCLUSIONS AND IMPLICATIONS

This study revealed differences in the effectiveness of multiple lineage-level diversification rate-based and growth-based prioritization measures in identifying potential for future transmission, via both empirical and simulated data approaches. Phylogenetic clustering of viral sequences provides a platform for prioritization of groups that aims to maximize the benefits of limited public health resources while still maintaining patient confidentiality. However, existing methods of prioritization are limited by their subjectivity and reliance on historical or linked data. Previous studies have demonstrated the advantages of prioritizing transmission clusters based on growth-based measures such as the square of cluster growth [16] and phylogenetic measures such as the terminal branch length [28]. In concordance with Moshiri *et al*. [28], we find phylogenetically derived measures, specifically those derived from lineage-level diversification rates, confer several advantages.

In the empirical BC dataset, measures based on phylogenetically derived lineage-level diversification rates demonstrated relatively similar ability to delineate prioritized clusters from less urgent clusters relative to growth-based measures overall. Simulated data further supported this, revealing a stronger relationship between the large majority of diversification rate-based measures and future cluster growth than seen for growth-based measures. The disparity in the strength of this relationship between diversification rate and growth-based measures became greater when the growth period under study was extended from only the next year to all future years remaining in the simulation period, suggesting that diversification rate-based measures are more closely associated with long-term growth. This confers an additional advantage to diversification rate-based measures, as a strong relationship with long-term growth could further maximize the downstream benefit of the public health resources distributed following prioritization.

Analyses of decreased sampling proportion revealed further advantages of diversification rate-based measures, which were more consistently robust to the effects of missing data than growth-based measures. The practical implications of this finding are important, as even the most well-sampled epidemics rarely come close to 100% sampling, so in order to be widely applicable, a prioritization measure needs to be robust to the effects of missing data in a wide range of epidemic circumstances. However, it should be noted that random downsampling will not provide an exact recapitulation of natural sampling bias, and the true effects of lower sampling proportion may differ from our findings. Moshiri *et al*. [28] similarly found the use of terminal branch lengths to be relatively robust to downsampling, which taken together with our findings perhaps suggests that phylogenetically derived measures, and in particular those weighted towards the present, are more robust to the effects of missing data overall. Another potential advantage of diversification rate measures is that in the case of larger clusters, they could be used to reveal subpopulations within a cluster in need of rapid intervention, even in cases where the cluster as a whole may not be prioritized.

An important limitation of lineage-level diversification rate is its sensitivity to sampling rate. Because diversification rate highlights lineages with increased recent branching, it is possible that lineages in well-sampled areas of the tree may demonstrate higher diversification rates than lineages in poorly sampled areas of the tree, regardless of the true transmission rate associated with each. Thus, the use of diversification rates in prioritization may demonstrate bias towards highlighting clusters of rapid detection rather than clusters of rapid transmission. Finally, although non-phylogenetic methods of both cluster inference and cluster prioritization have the advantage of being much less computationally demanding, we argue that the disadvantages of non-phylogenetic cluster inference [29–31] and non-phylogenetic growth-based prioritization outweigh this benefit, especially as increasing computational power [32] extends the feasibility of phylogenetic approaches.

Both empirical and simulated data revealed similar trends in the overall abilities of individual measures. Measures based on the most recent diversification rate(s) consistently performed considerably worse relative to other measures, further demonstrating the limited abilities of measures failing to capture long-term trends. In contrast, MaxDR, MeanDR, Top3MeanDR and Top5MeanDR offered the most consistency, achieving statistically significant separation between the urgent priority and remaining population of clusters in eight of the years studied. Although two growth-based measures (PrevYr5CasePlus and Prev3YrGrowth) meet this standard, they rely entirely on the existence of historical data, a shortcoming that could be eliminated by use of diversification rate-based measures. One such measure, Top5MeanDR, not only achieves statistically significant separation in 8 of the 10 years studied in the BC dataset, but also shows the strongest relationship with cluster growth across all simulated growth periods and parameter sets studied of all tested prioritization measures. This is in concordance with analyses conducted by McLaughlin *et al*. in 2019 [33], which found the mean of the top five log diversification rates in a geographic area to be a significant predictor of new HIV cases in BC. However, several other measures displayed consistent ability to delineate clusters based on priority status in addition to showing relatively strong relationships with future cluster growth, and as suggested by McLaughlin *et al*., it is likely that prioritization could be further optimized beyond the reaches of a single measure via a model combining multiple factors [33, 34]. Furthermore, as mentioned by Moshiri *et al*. [28], even a combined measure is unlikely to capture the full context of the epidemic, and measures that aim to improve the prioritization process should still be considered in the context of other supporting knowledge.

Overall, we find that diversification rate-based measures not only frequently outperform growth-based measures in their ability to identify groups with the highest potential for future growth, particularly long-term future growth, but are also less likely to be perturbed by missing data and remain free of need for historical data and subjective interpretation. In contrast, although growth-based measures can sometimes be equally effective as measures based on phylogenetic diversification rates, they have the potential to become misleading in cases of lower sampling proportion. In combination with phylogenetic clustering, phylogenetically derived lineage-level diversification rates can provide a simple, widely applicable and robust solution to focus prioritization of transmission clusters contributing the most to an ongoing epidemic.

## Supplementary Material

eoac026_Supplementary_Data

## Data Availability

Due to the confidential nature of population level HIV sequence data sets and the criminalization of HIV transmission in Canada, the HIV sequences and associated meta data will not be made publicly available. All other data are publicly available at the sources cited.
