# Supplementary material for: Phylogenetic prioritization of HIV-1 transmission clusters with viral lineage-level diversification rates
Source: Evol Med Public Health. 2022 Jul 22;10(1):305–15. doi: 10.1093/emph/eoac026 (PMC9311310; doi:10.1093/emph/eoac026)
Supplement: eoac026_Supplementary_Data [file eoac026_supplementary_data.pdf]

Supplementary Materials

Optimization of FAVITES Parameters

Simulations were performed using the FAVITES framework (1). Initial estimates of parameter choices were iteratively refined to concurrently optimize the number of output sequences (Figure S11), phylogeny branch lengths and structure (Figure S12-14), the diversification rate (Figure S15) distribution and the transmission cluster size distribution (Figure S16) to best mimic the BC epidemic. Each variation on the base parameter set changes only one parameter, either the number of seed individuals (and corresponding contact network size), the length of time to begin ART, or the number of contacts each individual has within the contact network. A full list of parameter choices can be found in Table S1.

Parameters not mentioned going forward can be assumed to follow the parameter choices and justification seen in Moshiri *et al*. (2019) (1). In all parameter sets, the contact network was generated under a Barabási-Albert model, which generates scale-free networks reflective of the connectivity of social or sexual networks (1-3). The three values chosen for the number of seed individuals were selected to represent the sum of undiagnosed plus untreated diagnosed individuals (i.e. not virologically suppressed infected population), where the diagnosed population yet to begin treatment was assumed to be approximately 15% of the total diagnosed population (4). This assumption is based on values estimated for 2013, as this marks the middle of the simulation period. The number of seed individuals was selected to reflect epidemic conditions at the start of the simulation period (2009), so that the simulation would be able to progress similarly to the real epidemic scenario over the following ten years. Contact network sizes corresponding to the chosen seed values, and the three values chosen for the expected degree of connection of each contact network node were selected based on 2008 estimates

regarding the BC epidemic (5). Seed selection from the contact network was conducted at random.

Transmissions occurred under an adapted version of the HIV-ART model described by Granich *et al*. (6). Expected time to transition from an untreated state to a treated state was set to either 0.5, 1 or 2 years. Expected time to transition from a treated state back to an untreated state was set to 10 years, to represent BC's relatively high rate of ART retention (7).

In the BC dataset, which has been estimated to achieve approximately 75% sampling of the total prevalent population (4) 3,094 new sequences were collected in the 2009-2018 period. Thus, transmission rates were scaled such that the number of new infections generated during the simulation period for the lowest number of seeds was equal to the sum of the number of seeds plus approximately 3,868 new infections (Figure S11).

A subset of the BC epidemic phylogeny containing only subtype B sequences collected during 2009 and onwards was used to infer parameters related to sequence evolution and mutation rates. Mutation rates were sampled from a truncated $(0, \infty)$ normal distribution. LSD2 (8) was used to determine an initial estimate of the mutation rate, which was then used in combination with patristic distance distributions to refine the location and scale parameters of the mutation rate distribution such that Jensen-Shannon Divergence (JSD) scores comparing the real and simulated patristic distance distributions were minimized (Figure S12). The opposing subset of the BC phylogeny containing only sequences collected before 2009 was used to estimate the time to most recent common ancestor (tMRCA), as this parameter is used to build the seed tree of individuals already infected at time zero. Using LSD2, the tMRCA from the end of 2008 was estimated to be 1946. The speciation rate of the seed tree was optimized to match the accumulation of lineages over time seen in the BC dataset before 2009 (Figure S17). Potential

rate functions demonstrating exponential decay were scaled for comparison in TreeSAP and the best match was confirmed to be appropriate via visual evaluation of similarity between the median lineage through time (LTT) plot of 20 FAVITES replicates versus the BC LTT plot. Sequence evolution was set to occur under the generalized time-reversible (GTR) substitution model with gamma rate heterogeneity and the associated parameters were inferred from the post-2008 BC alignment using IQ-TREE v1.61 (9).

Sampling was done at the time of ART initiation, meaning that 100% sampling indicates 100% of diagnosed cases, not 100% of infections. Each fully sampled sequence dataset was also randomly downsampled by year to 75%, 50% and 25% of diagnoses to investigate the impact of missing data. In order to account for the variation between FAVITES runs, 20 replicates were run for each of the seven parameter sets.

Initial FAVITES parameters were selected based on the literature or inferred from the BC dataset and optimized iteratively in order to achieve a closer match to the characteristics of the BC data. As there are many characteristics to consider in tandem, some were prioritized over others. In particular, the difference between the distribution of diversification rates in the real data versus the simulated data, quantified by JSD score, was minimized (Figure S15), as the prioritization measures under study rely heavily on these values. Other characteristics considered in the optimization of parameters were the tree structure, branch lengths, root-to-tip distance, and the distribution of transmission cluster sizes (Figures S5-7). Since FastTree ignores nucleotide ambiguities, neither ambiguities in the empirical dataset nor those generated by FAVITES were considered in the comparison.

Despite efforts to capture the characteristics of the BC epidemic in our simulations, we recognize that by drawing from fixed distributions and models, our simulation parameters

assume a certain level of predictability that may not always be in line with fluctuations and exceptions occurring in the real world, thus resulting in data that approximates real circumstances in general rather than providing an exact replica truth. It should also be noted that the list of prioritization measures studied here is not exhaustive and study of additional measures may allow further optimization of prioritization.

Supplementary Figures

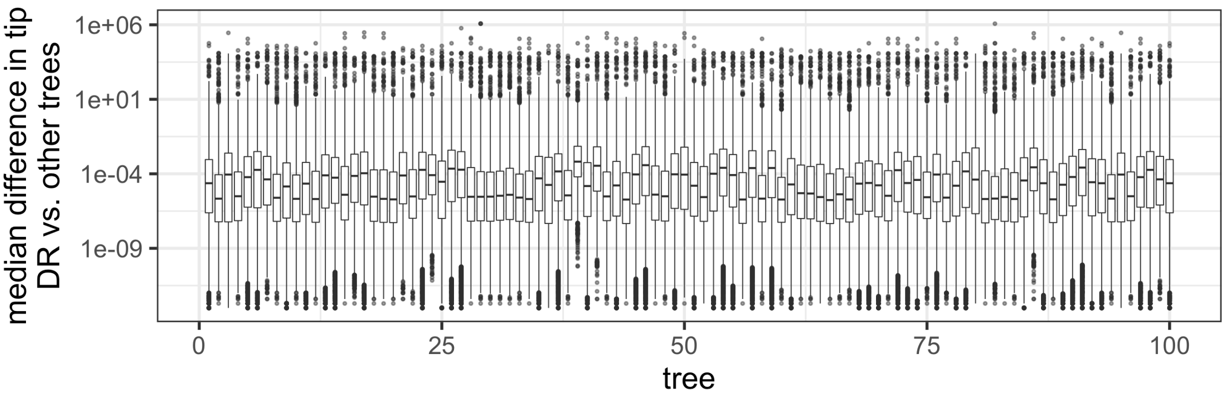

Figure S1. Distribution of the median difference diversification rates across trees for each tip. Boxplots represent the median and IQR of the median difference in tip diversification rate in a given tree versus all other trees. In general, the median difference in diversification rate was very small across the 100-tree distribution, but some tips showed greater variation.

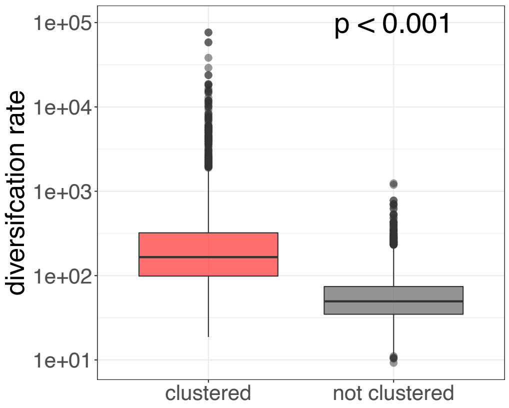

Figure S2. Boxplot comparing the median and IQR of the diversification rates from all clustered tips versus all non-clustered tips. Significance of the difference between the two groups was assessed via a Mann-Whitney test. The increased diversification rates within clusters suggest potential for more rapid transmission within these groups, and further justify their position as a public health focus.

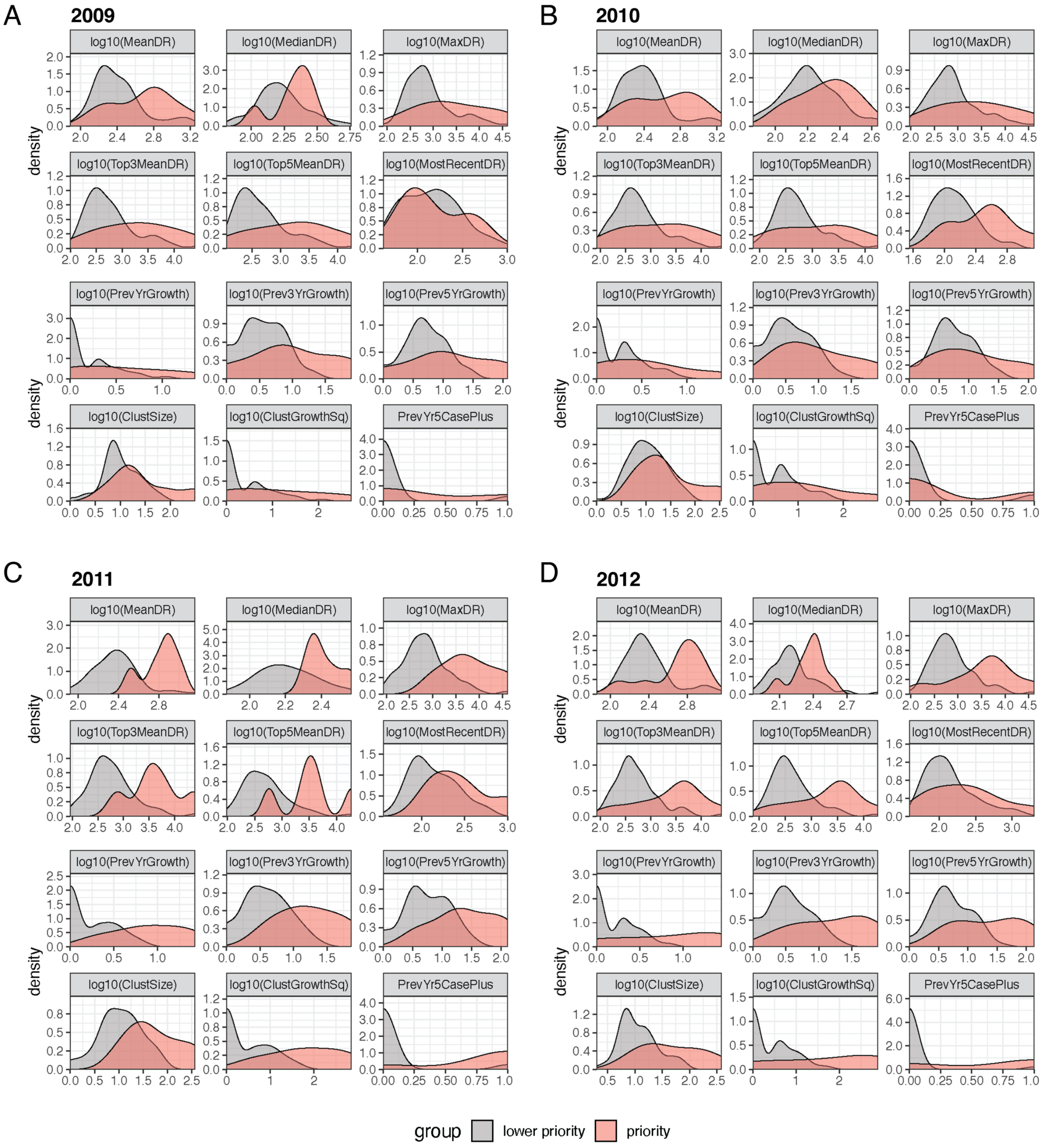

A
2009
B
2010
C
2011
D
2012
log10(MeanDR)
log10(MedianDR)
log10(MaxDR)
log10(Top3MeanDR)
log10(Top5MeanDR)
log10(MostRecentDR)
log10(PrevYrGrowth)
log10(Prev3YrGrowth)
log10(Prev5YrGrowth)
log10(ClustSize)
log10(ClustGrowthSq)
PrevYr5CasePlus
density
group
lower priority
priority

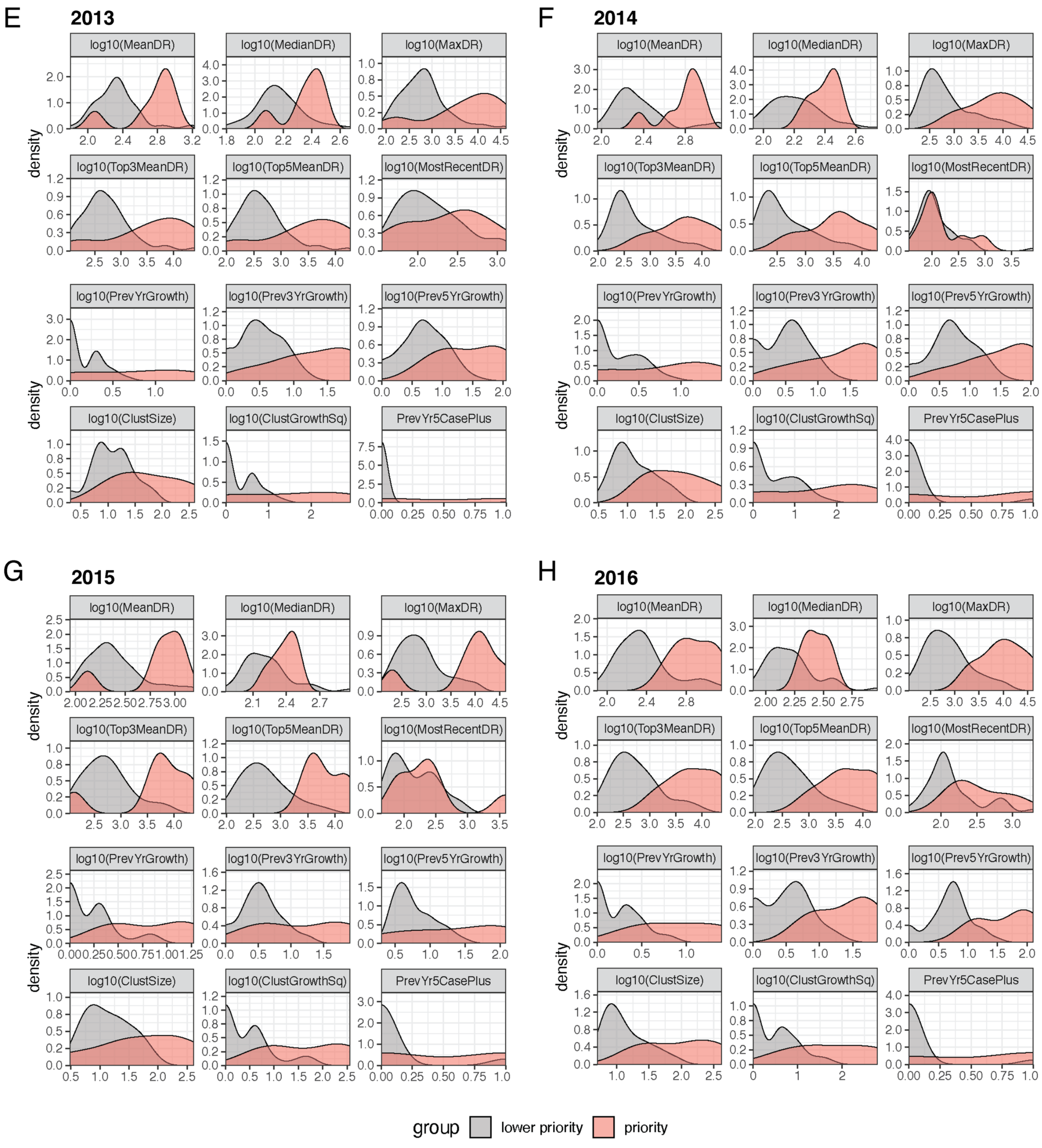
E
2013
F
2014
G
2015
H
2016
log10(MeanDR)
log10(MedianDR)
log10(MaxDR)
log10(Top3MeanDR)
log10(Top5MeanDR)
log10(MostRecentDR)
log10(PrevYrGrowth)
log10(Prev3YrGrowth)
log10(Prev5YrGrowth)
log10(ClustSize)
log10(ClustGrowthSq)
PrevYr5CasePlus
density
group
lower priority
priority

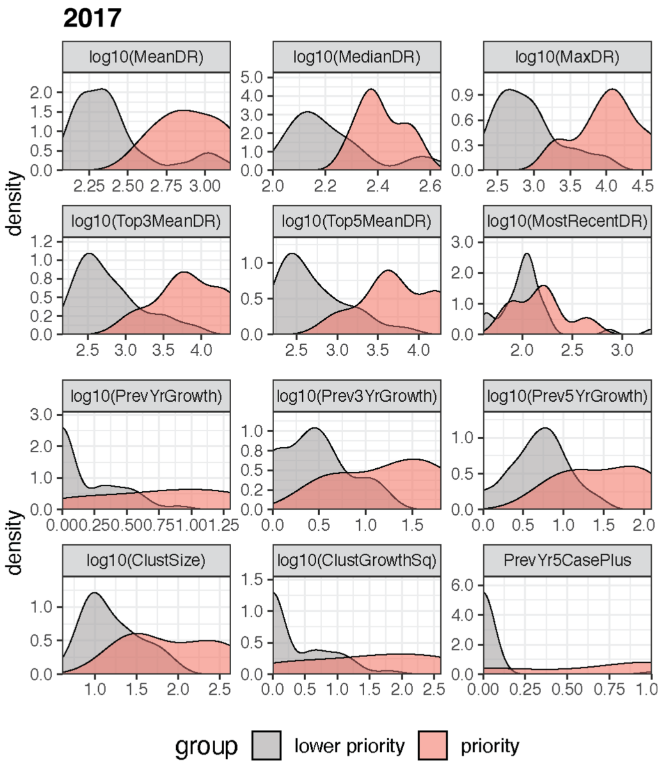

Figure S3. Density plots showing the difference in diversification rate-based measures or growth-based measures in 2009-2017 (A-I) between clusters defined as “priority” by the current public health protocol for immediate intervention and the remainder of the clusters to next be addressed, marked here for the purpose of comparison as “lower priority”. Only clusters that had newly diagnoses cases added in the previous year are shown. Infinite values created by the log10 transformation were forced to 0 for visualization purposes.

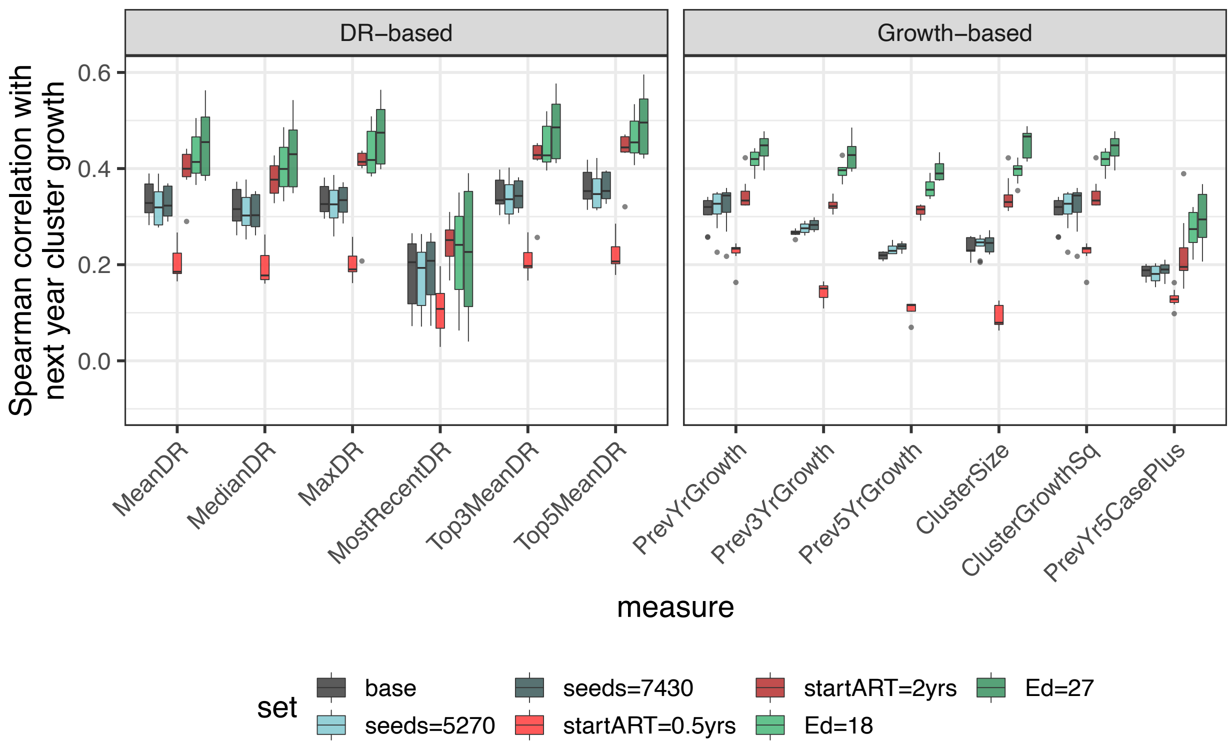

Figure S4. Spearman correlations between a given prioritization measures and future cluster growth in the next year. Each box represents all growth periods from 2009-2018 for a given prioritization measure and parameter set, showing the median and IQR. Abbreviations are as defined in Figure 2 and 3. In general, diversification rate-based measures showed slightly stronger correlations with growth in the next year growth-based measures.

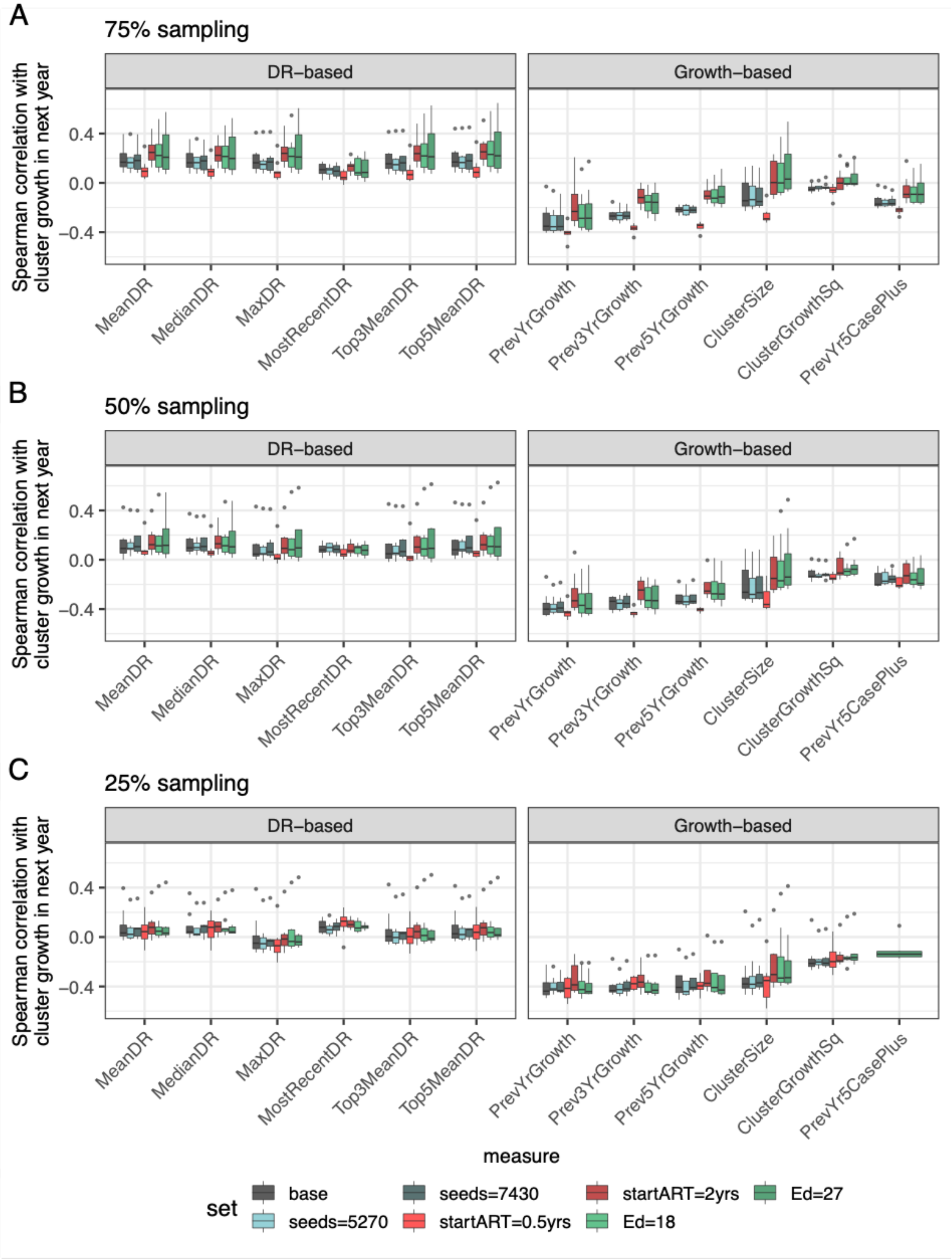

Figure S5. Boxplots comparing the median and IQR of the Spearman correlations between a given prioritization measure and cluster growth in the next year at A) 75% sampling, B) 50% sampling, and C) 25% sampling. Abbreviations are as defined in Figure 2 and 3. Only one parameter set is shown for PrevYr5CasePlus in panel C because correlation cannot be appropriately calculated when the binary value of this measure is consistently zero, a phenomenon that becomes increasingly more likely as the dataset is downsampled. As sampling

proportion decreases, the positive relationship between diversification rate-based measures and growth in the next year weakens and, in some cases, becomes almost non-existent. However, the correlation between growth-based measures and next year growth not only experiences weakening, but also reversal.

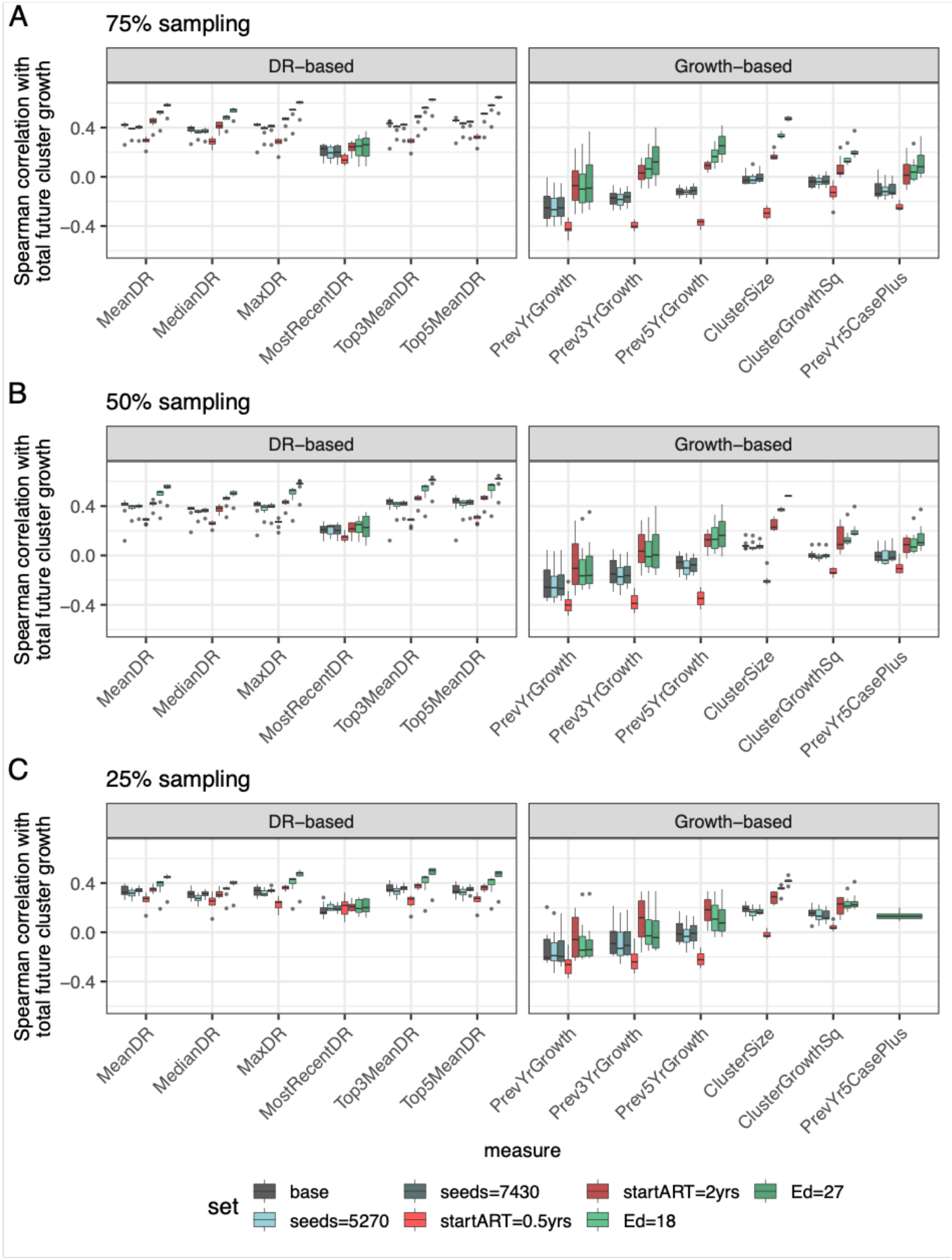

Figure S6. Boxplot comparing the median and IQR of the Spearman correlations between a given prioritization measures and total future cluster growth at A) 75% sampling, B) 50% sampling and C) 25% sampling. Abbreviations are as defined in Figure 2 and 3. Diversification rate-based measures demonstrate very little change in correlation with future growth as sampling proportion is decreased, and although some growth-based measures such as previous year growth again show inverted correlations, other measures such as cluster size and cluster growth squared

retain a relatively similar effect size, particularly when the time to start ART or the number of contacts is increased from the base parameter set.

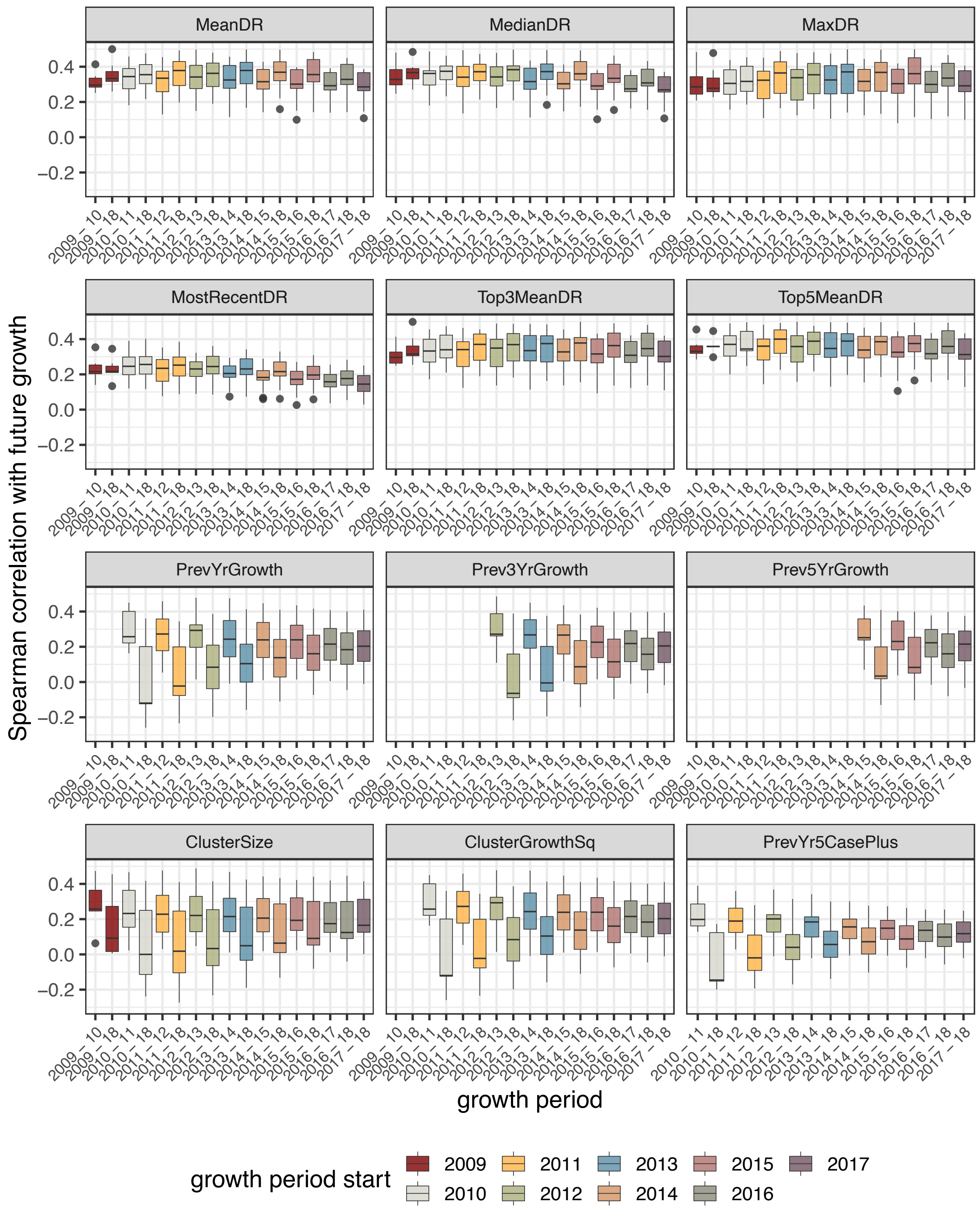

Figure S7. Spearman correlation between each prioritization measure and cluster growth. Growth periods end either in the year following prioritization measure calculation or the final year of the simulation (anywhere from one to nine years after prioritization, depending on the starting year). Boxes show the median and IQR of the effect sizes for each growth period, across all parameter sets. Boxes are colored based on the starting year of the growth period, ie. the year of prioritization. Abbreviations are as defined in Figure 2. Diversification rate measures showed a small increase in effect size as the growth period was extended from the next year to the full simulation period, but the opposite trend was seen for growth-based measures.

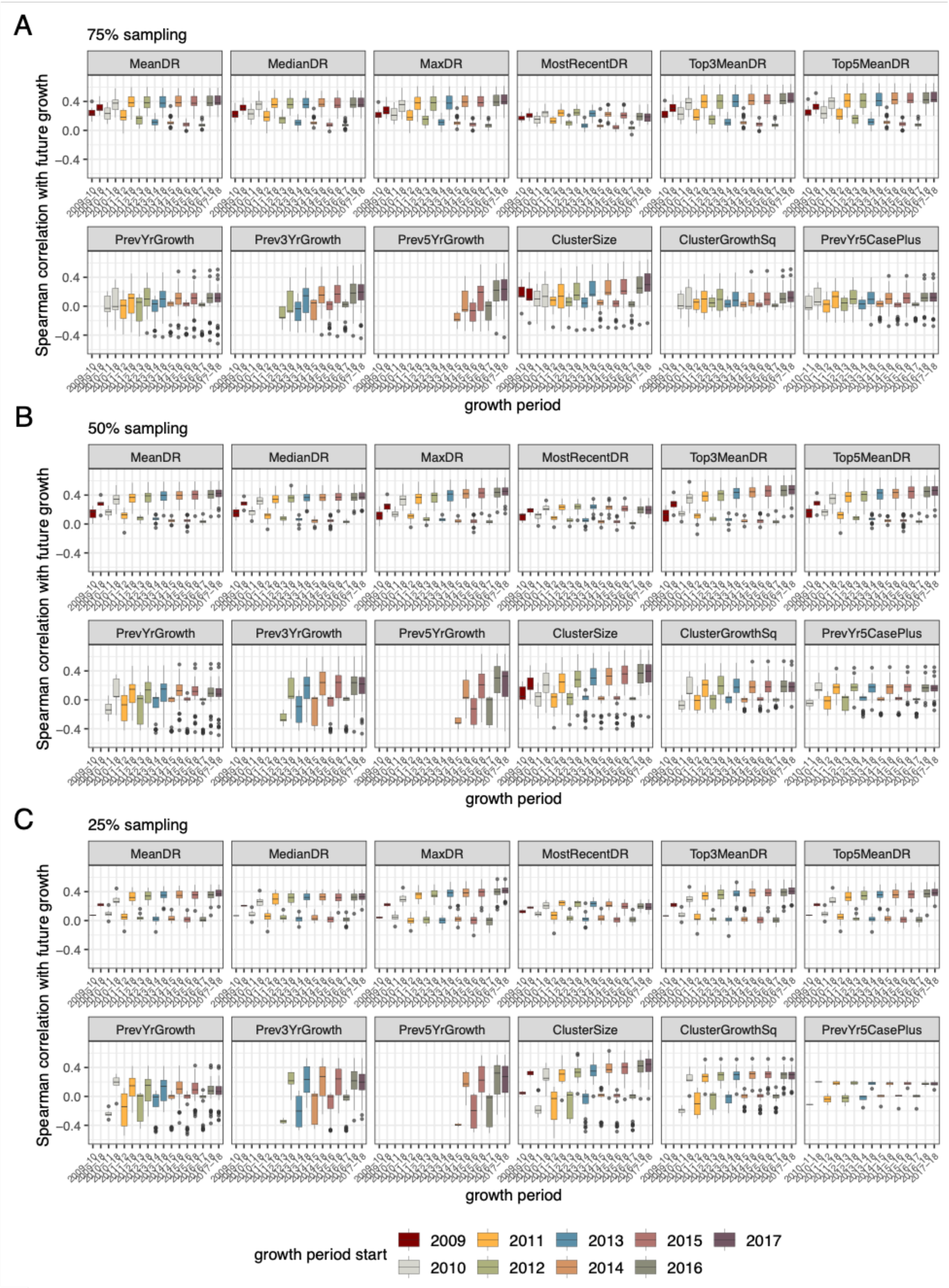

Figure S8. Mean Spearman correlation between each prioritization measure with future cluster growth, sampled at A) 75%, B) 50% and C) 25%. Growth periods end either in the year following prioritization measure calculation or the final year of the simulation (anywhere from one to nine years after prioritization, depending on the starting year). Boxes show the median

and IQR of the effect sizes for each growth period, across all parameter sets. Boxes are colored based on the starting year of the growth period, ie. the year of prioritization. Abbreviations are as defined in Figure 2. The corresponding fully sampled results can be found in Figure S6.

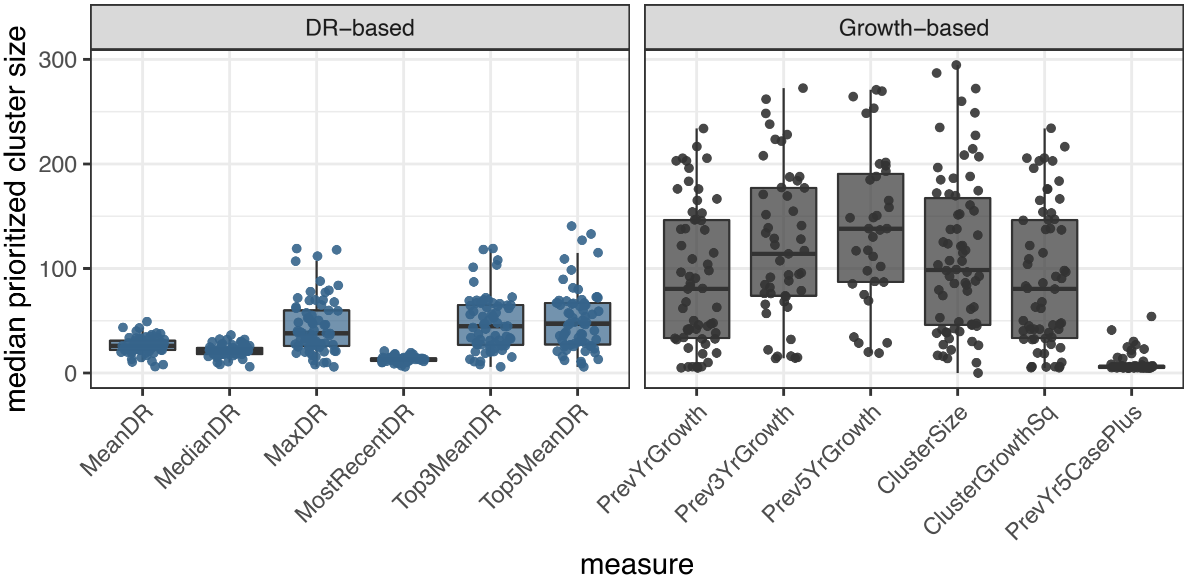

Figure S9. Boxplot comparing the median and IQR of the median size of the clusters prioritized by each prioritization measure. Abbreviations are as defined in Figure 2. In most cases, median cluster sizes are smaller for clusters prioritized by diversification rate measures than for clusters prioritized by growth-based measures.

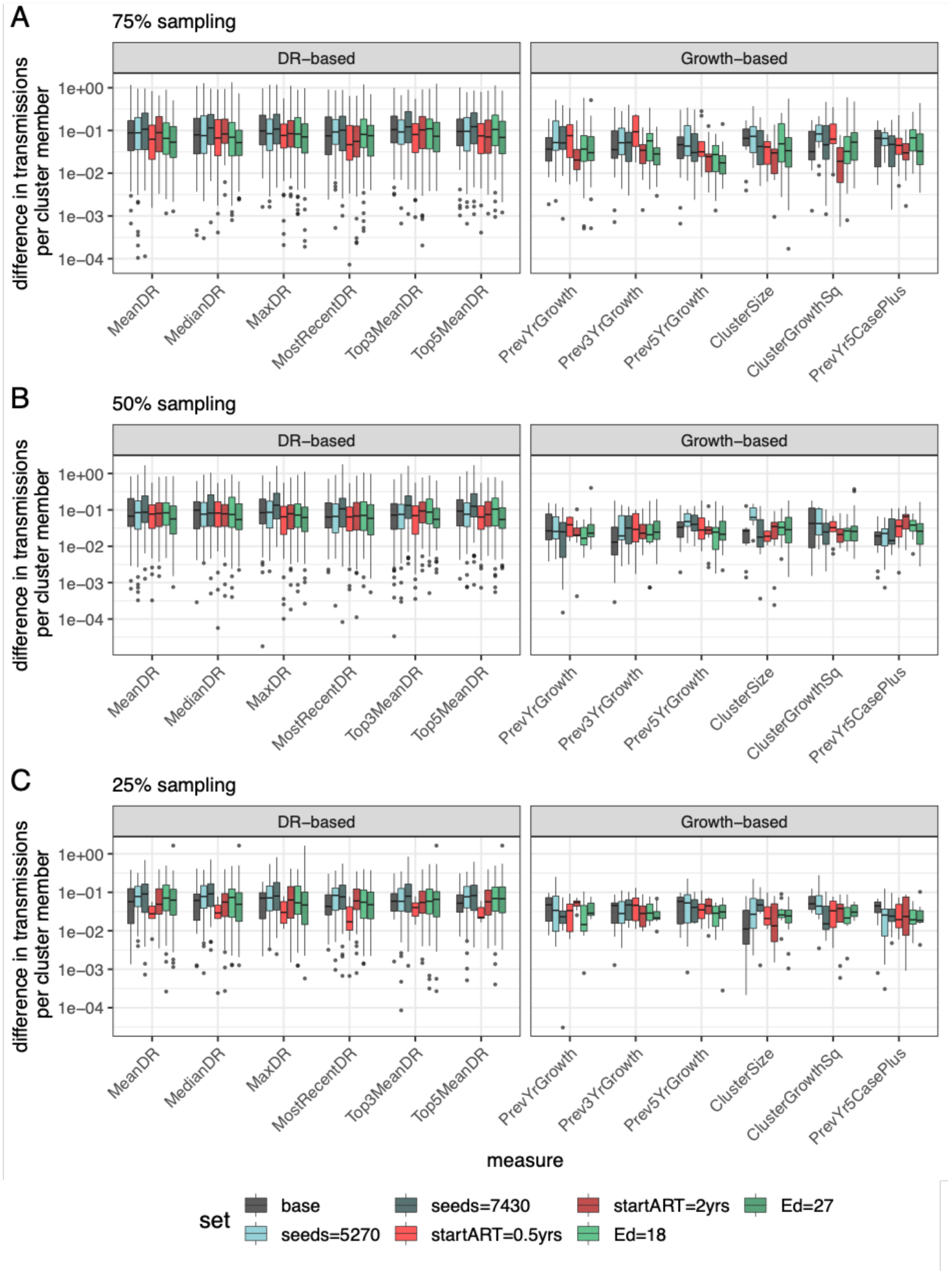

Figure S10. Boxplot comparing the median and IQR of the difference in mean direct transmissions per cluster member between the top prioritized clusters, up to inclusion of 100 individuals, according to a given prioritization measure and a random sample of lower-ranking clusters containing the same number of individuals, at A) 75% sampling, B) 50% sampling and C) 25% sampling. Abbreviations are as defined in Figure 2 and 3. Decreases in sampling

proportion generally result in maintenance of difference in transmissions for diversification rate measures (with the exception of one parameter set at 25% sampling) while the growth-based measures undergo a decline.

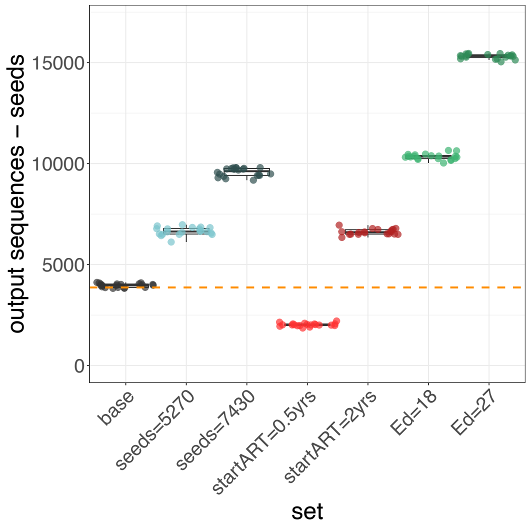

Figure S11. Boxplot showing the median and IQR of the number of sequences output by each simulation parameter set, after subtraction of seed individuals. The orange dashed line indicates the target number of output sequences. As. intended, the chosen parameter sets resulted in a range of outcomes in number of output sequences, some a closer match to the empirical BC data, and some more representative of other epidemics with different characteristics. Parameter set abbreviations are as defined in Figure 3.

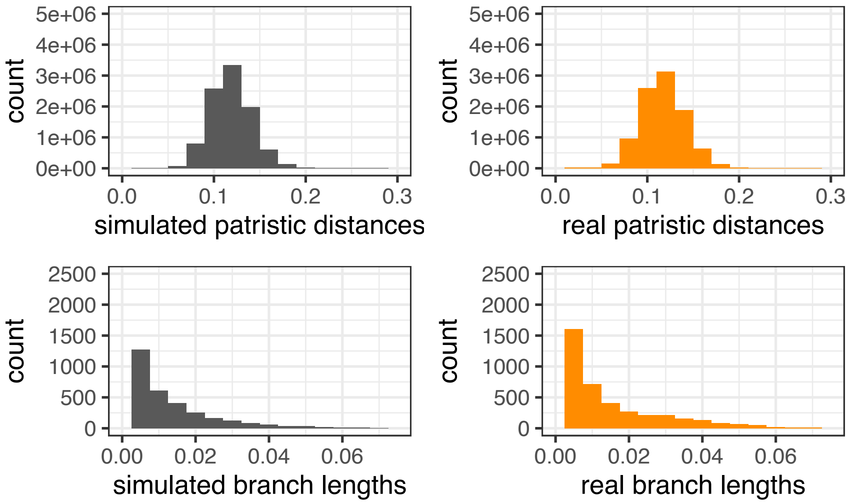

Figure S12. Comparison of simulated to real patristic distances and branch lengths, shown for one replicate of the base parameter set. Differences between real and simulated patristic distances and branch length distributions were iteratively minimized based on Jensen-Shannon Divergence (JSD) scores.

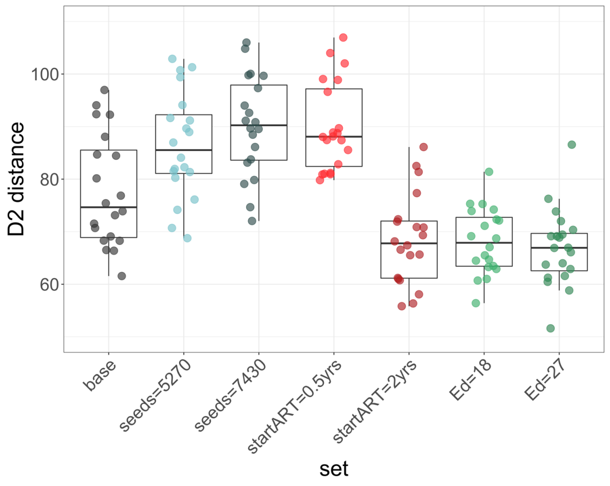

Figure S13. Comparison of simulated tree structure to real tree structure. Difference was quantified by D2 distance (10) across 20 replicates. Boxplots show the median and IQR of the D2 distance measure. Parameter set abbreviations are as defined in Figure 3.

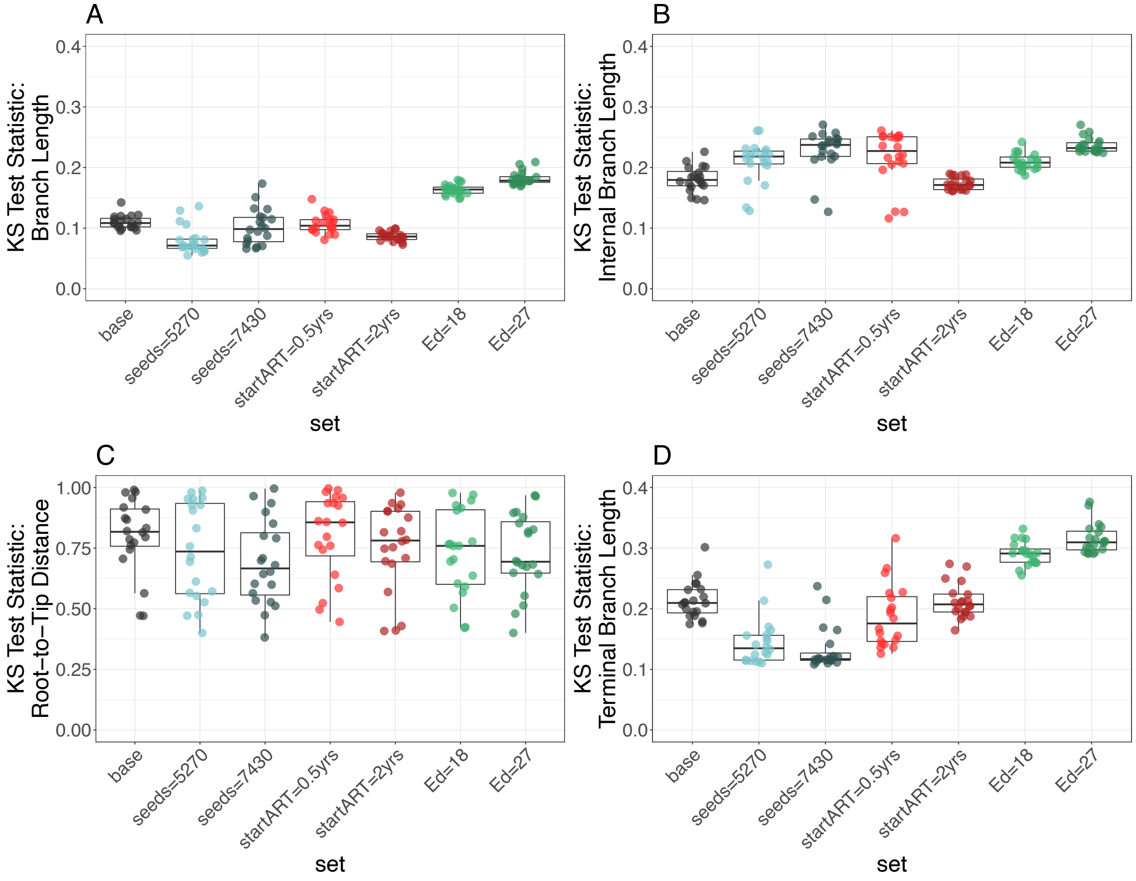

Figure S14. Boxplots showing the median and IQR of the Kolmogorov-Smirnov test statistic evaluating the similarity of simulated versus empirical A) branch lengths, B) internal branch lengths, C) root-to-tip distance and D) terminal branch lengths. As intended, the chosen parameter sets resulted in a range of outcomes in number of output sequences, some a closer

match to the empirical BC data, and some more representative of other epidemics with different characteristics. Parameter set abbreviations are as defined in Figure 3.

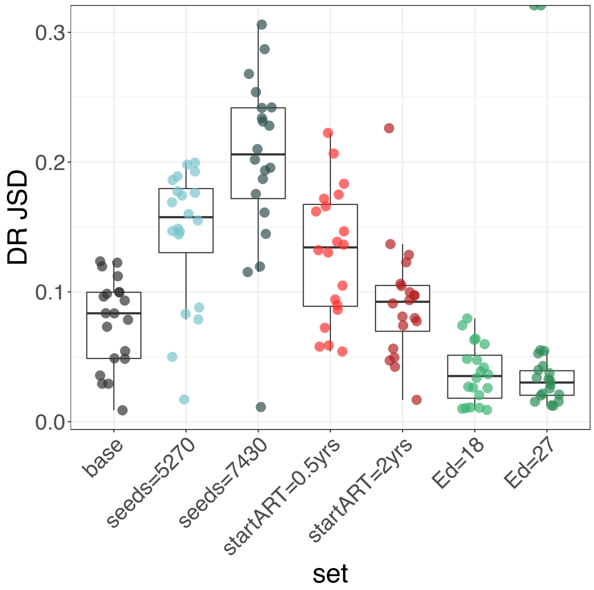

Figure S15. Boxplot showing the median and IQR of the Jensen-Shannon Divergence (JSD) scores between the simulated and real distribution of diversification rates across 20 replicates. Differences between real and simulated lineage-level diversification rates were iteratively minimized based on JSD scores. Parameter set abbreviations are as defined in Figure 3.

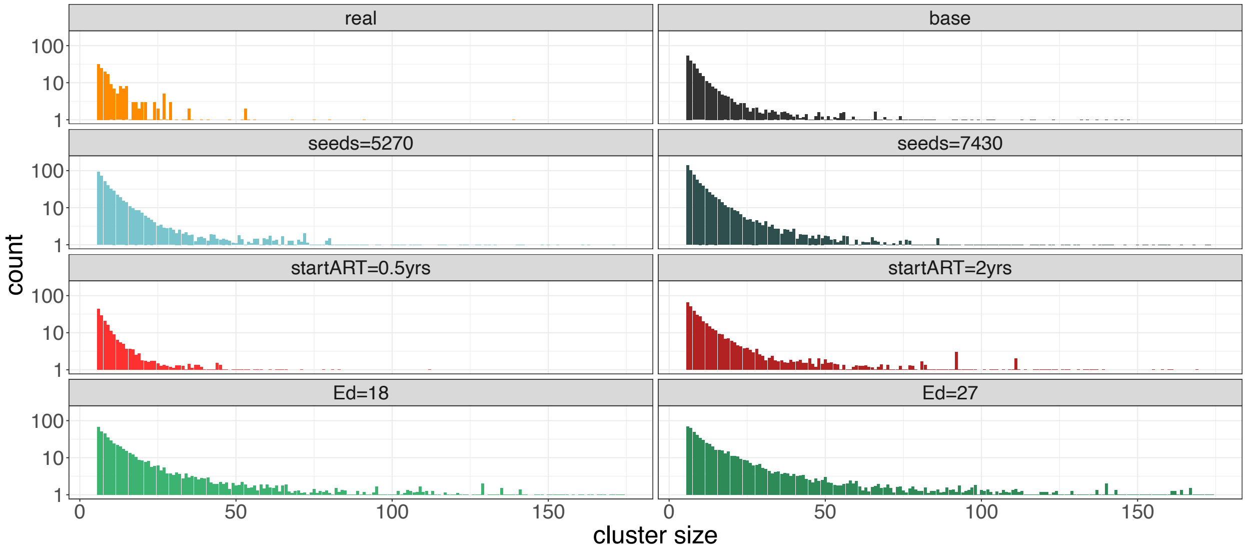

Figure S16. Cluster size distributions for the final year of each simulated parameter set, compared to cluster size distributions from empirical data from the same year. Counts of cluster sizes represent the mean counts across 20 simulation replicates. As intended, the chosen parameter sets resulted in a range of cluster size distribution outcomes, some a closer match to the empirical BC data, and some more representative of epidemics with different characteristics. Parameter set abbreviations are as defined in Figure 3.

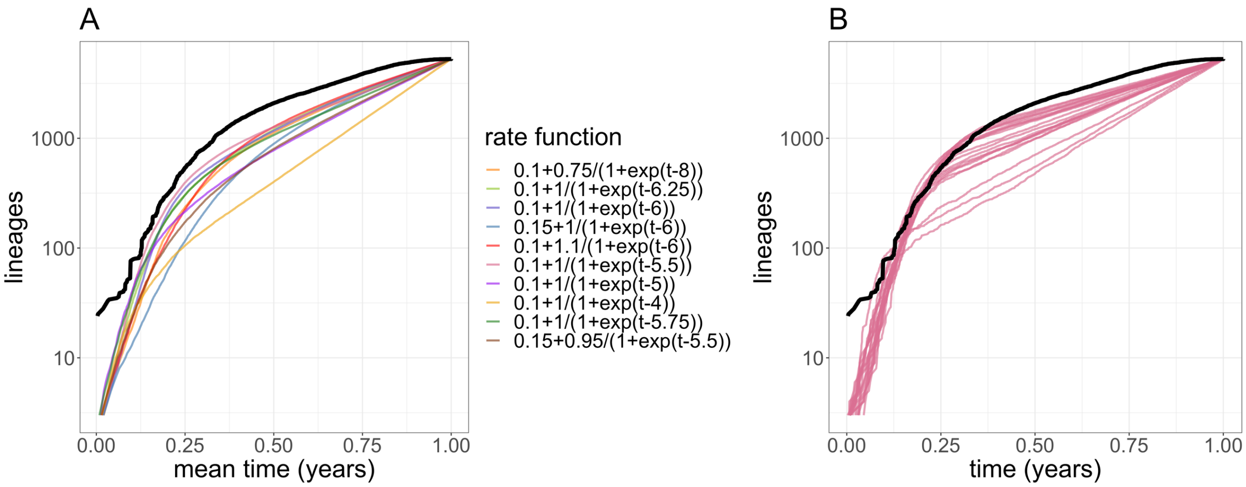

Figure S17. A) Comparison of the mean accumulation of lineages through time (LTT) for several possible rate functions, scaled to a 1 year time period in TreeSAP. The LTT line representing the real data is shown in black. B) Accumulation of lineages through time resulting from simulation in FAVITES for the selected rate function, shown for 20 replicates. The most appropriate rate function was selected via visual evaluation of similarity between the median LTT line of 20 FAVITES replicates versus the BC LTT line.

Supplementary Tables

Table S1. FAVITES simulation parameters. Parameters in bold were varied across parameter sets.

**Parameter**	**Value**
ContactNetworkGenerator	Barabasi-Albert
**num_cn_nodes**	(26746, 45322, 63898)
**num_edges_from_new**	(9, 18, 27)
SeedSelection	Random
**num_seeds**	(3110, 5270, 7430)
SeedSequence	VirusNonHomYuleHeightGTRGamma
seed_height	62
viral_sequence_type	HIV1-B-DNA-POL-LITTLE
seed_speciation_rate_func	0.1+1/(1+exp(t-5.5))
TransmissionTimeSample	HIVARTGranichGEMF
hiv_a1_to_a2	4.333333
hiv_a1_to_i1	0.096
hiv_a2_to_i2	0.096
**hiv_i1_to_a1**	(0.5, 1, 2)
hiv_i1_to_i2	8.666667
**hiv_i2_to_a2**	(0.5, 1, 2)
hiv_s_to_i1_by_a1	0.002
hiv_s_to_i1_by_i1	0.04
hiv_s_to_i1_by_i2	0.008
end_time	10
TreeUnit	Truncated Normal
tree_mutation_loc	0.0003
tree_mutation_scale	0.0008
tree_mutation_min	0
tree_mutation_max	Inf
SequenceEvolution	GTRGammaSeqGen
GTR state frequencies	[A: 0.395, C:0.171, T: 0.211, G:0.222]
GTR transition rates	[λ_{AC}: 1.75637, λ_{AG}: 8.32038, λ_{AT}: 0.629219, λ_{CG}: 0.71545, λ_{CT}: 8.32038, λ_{GT}: 1]
seqgen_gamma_shape	0.4237896
NodeEvolution	VirusTreeSimulator
vts_model	logistic
vts_no	1
vts_growthRate	2.851904
vts_t50	-2
TimeSample	GranichFirstART
NumTimeSample	Once

References

1. Moshiri N, Ragonnet-Cronin M, Wertheim JO, et al.; FAVITES: simultaneous simulation of transmission networks, phylogenetic trees and sequences. *Bioinformatics* 2019;**35**(11):1852-1861. doi: 10.1093/bioinformatics/bty921.
2. Barabasi AL, Albert R; Emergence of scaling in random networks. *Science* 1999;**286**(5439):509-12. doi: 10.1126/science.286.5439.509.
3. Hamilton DT, Handcock MS, Morris M; Degree distributions in sexual networks: a framework for evaluating evidence. *Sex Transm Dis* 2008;**35**(1):30-40. doi: 10.1097/olq.0b013e3181453a84.
4. Lima VD, Brumme ZL, Brumme C, et al.; The Impact of Treatment as Prevention on the HIV Epidemic in British Columbia, Canada. *Curr HIV/AIDS Rep* 2020;**17**(2):77-87. doi: 10.1007/s11904-020-00482-6.
5. HIV and Sexually Transmitted Infections Report. Clinical Prevention Services, British Columbia Centre for Disease Control, 2010.
6. Granich RM, Gilks CF, Dye C, et al.; Universal voluntary HIV testing with immediate antiretroviral therapy as a strategy for elimination of HIV transmission: a mathematical model. *Lancet* 2009;**373**(9657):48-57. doi: 10.1016/S0140-6736(08)61697-9.
7. Nosyk B, Montaner JSG, Colley G, et al.; The cascade of HIV care in British Columbia, Canada, 1996-2011: a population-based retrospective cohort study. *Lancet Infect Dis* 2014;**14**(1):40-49. doi: 10.1016/S1473-3099(13)70254-8.
8. To TH, Jung M, Lycett S, et al.; Fast Dating Using Least-Squares Criteria and Algorithms. *Syst Biol* 2016;**65**(1):82-97. doi: 10.1093/sysbio/syv068.
9. Nguyen LT, Schmidt HA, von Haeseler A, et al.; IQ-TREE: a fast and effective stochastic algorithm for estimating maximum-likelihood phylogenies. *Mol Biol Evol* 2015;**32**(1):268-74. doi: 10.1093/molbev/msu300.
10. Colijn C, Plazzotta G; A Metric on Phylogenetic Tree Shapes. *Syst Biol* 2018;**67**(1):113-126. doi: 10.1093/sysbio/syx046.
